# An *in vivo* systemic massively parallel platform for deciphering animal tissue-specific regulatory function

**DOI:** 10.3389/fgene.2025.1533900

**Published:** 2025-04-09

**Authors:** Ashley R. Brown, Grant A. Fox, Irene M. Kaplow, Alyssa J. Lawler, BaDoi N. Phan, Lahari Gadey, Morgan E. Wirthlin, Easwaran Ramamurthy, Gemma E. May, Ziheng Chen, Qiao Su, C. Joel McManus, Robert van de Weerd, Andreas R. Pfenning

**Affiliations:** ^1^ Ray and Stephanie Lane Department of Computational Biology, Carnegie Mellon University, Pittsburgh, PA, United States; ^2^ Neuroscience Institute, Carnegie Mellon University, Pittsburgh, PA, United States; ^3^ Department of Biological Sciences, Carnegie Mellon University, Pittsburgh, PA, United States; ^4^ Medical Scientist Training Program, University of Pittsburgh School of Medicine, Pittsburgh, PA, United States

**Keywords:** machine learning, aav, *in vivo*, enhancer, PHP.eB, brain, tissue specific, transcriptional regulation

## Abstract

**Introduction:** Transcriptional regulation is an important process wherein non-protein coding enhancer sequences play a key role in determining cell type identity and phenotypic diversity. In neural tissue, these gene regulatory processes are crucial for coordinating a plethora of interconnected and regionally specialized cell types, ensuring their synchronized activity in generating behavior. Recognizing the intricate interplay of gene regulatory processes in the brain is imperative, as mounting evidence links neurodevelopment and neurological disorders to non-coding genome regions. While genome-wide association studies are swiftly identifying non-coding human disease-associated loci, decoding regulatory mechanisms is challenging due to causal variant ambiguity and their specific tissue impacts.

**Methods:** Massively parallel reporter assays (MPRAs) are widely used in cell culture to study the non-coding enhancer regions, linking genome sequence differences to tissue-specific regulatory function. However, widespread use in animals encounters significant challenges, including insufficient viral library delivery and library quantification, irregular viral transduction rates, and injection site inflammation disrupting gene expression. Here, we introduce a systemic MPRA (sysMPRA) to address these challenges through systemic intravenous AAV viral delivery.

**Results:** We demonstrate successful transduction of the MPRA library into diverse mouse tissues, efficiently identifying tissue specificity in candidate enhancers and aligning well with predictions from machine learning models. We highlight that sysMPRA effectively uncovers regulatory effects stemming from the disruption of MEF2C transcription factor binding sites, single-nucleotide polymorphisms, and the consequences of genetic variations associated with late-onset Alzheimer‘s disease.

**Conclusion: **SysMPRA is an effective library delivering method that simultaneously determines the transcriptional functions of hundreds of enhancers in vivo across multiple tissues.

## Introduction

Transcriptional regulation, a process in which non-coding enhancer sequences play a major role, is a key component of specifying both cell type identity and phenotypic diversity (King and Wilson, 1975; Wray, 2007; Pennacchio et al., 2013; Cheng et al., 2014). In neural tissue, gene regulatory processes are essential for organizing the range of highly interconnected and regionally specialized cell types that must synchronize their activity to produce behavior (Goodman and Bonni, 2019). Insights from advancements reveal that transcription is largely regulated by enhancers, distal non-coding sequences that are highly tissue-specific relative to proximal promoters (Roadmap et al., 2015). Progress in experimental technologies has now enabled the direct profiling of open chromatin, a component of the “epigenomic” or gene regulatory landscape, both at the tissue and individual cell type levels (Buenrostro et al., 2013; Buenrostro et al., 2015; Mo et al., 2015; Lawler et al., 2020; Bryois et al., 2018). Despite this progress, it is noteworthy that numerous open chromatin regions lack the capability to activate transcription (Hrvatin et al., 2019; Singh et al., 2021; Glaser et al., 2021; Lawler et al., 2022).

**FIGURE 1:**
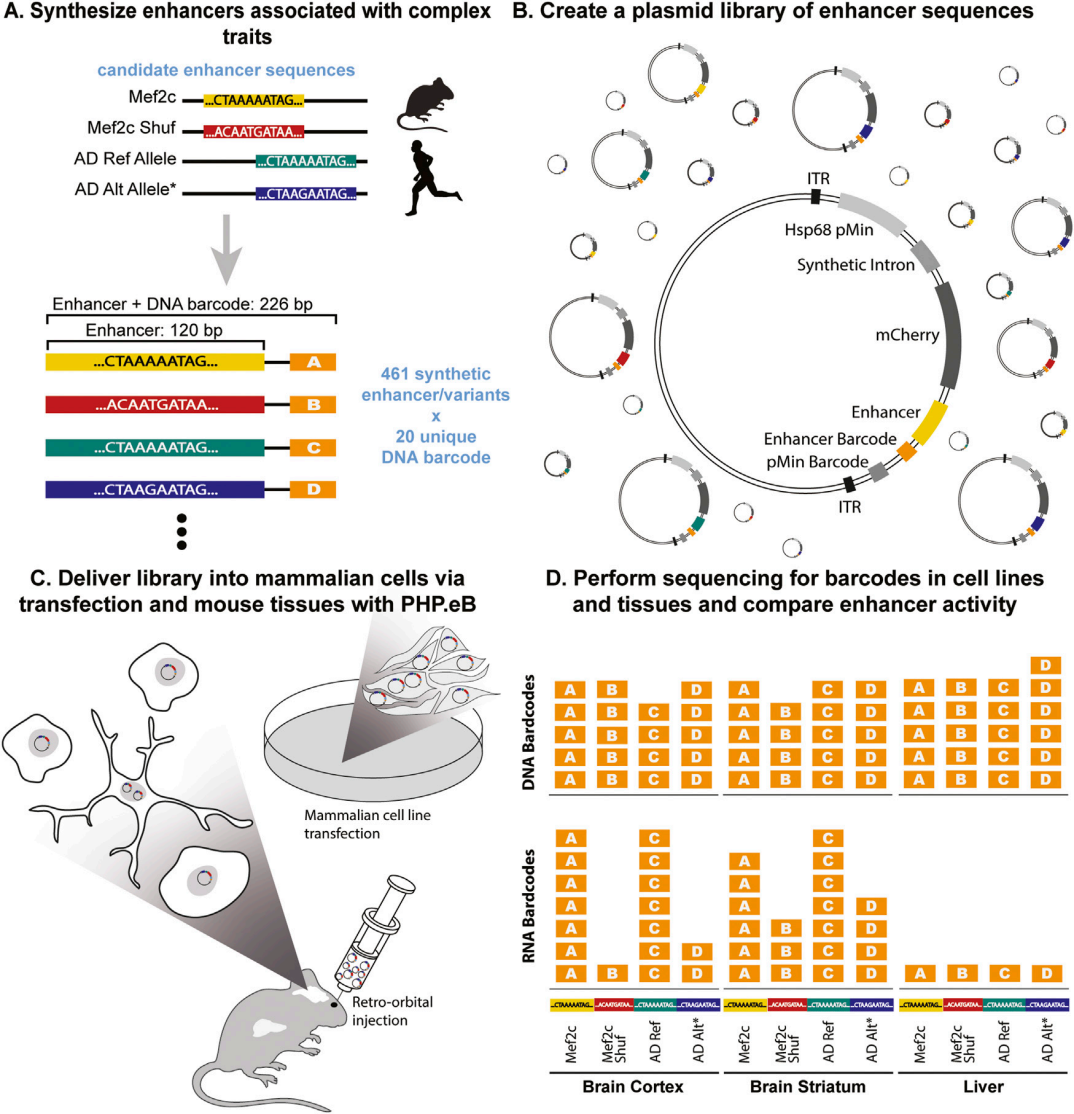
SysMPRA tests effects of transcription factor binding and single nucleotide variations on transcriptional regulation. **(A)** The library is designed to study complex traits consisting of 461 enhancers and variants, each with 20 unique barcodes (i.e., MEF2C motifs and shuffled versions of these motifs, as well as reference and alternative alleles for AD-associated SNPs). **(B)** The oligos are synthesized and cloned into plasmids containing a minimal promoter (Hsp68 pMin), a synthetic intron, mCherry, and inverted terminal repeats (ITRs) that enable recombination into AAV genomes. **(C)** The plasmid library is packaged into the PHP. eB AAV serotype and delivered into a mouse via retro-orbital injection or transfected into mammalian cells. **(D)** The activity of candidate enhancers in multiple brain regions and tissues is measured using DNA and RNA levels of the barcodes.

The arrival of high-throughput reporter assays (Sharon et al., 2012) has facilitated the experimental assessment of candidate enhancers, including open chromatin regions, for their ability to activate transcription. A prominent technology in this field is the massively parallel reporter assay (MPRA) (Kwasnieski et al., 2012; Melnikov et al., 2012; Kheradpour et al., 2013; Nguyen et al., 2016; Tewhey et al., 2016; Abell et al., 2022). This approach entails generating a library of numerous distinct plasmids, each incorporating a custom-synthesized candidate enhancer that controls the expression of one or more unique barcodes in conjunction with a minimal promoter (Figure 1A). Multiple approaches can be used to construct these MPRA libraries, including using enhancer capture involving the selection of open chromatin regions through profiling in the relevant cell lines (Wang et al., 2018; Arnold et al., 2013; Shen et al., 2016). These studies show the assessment of expression from distinctive plasmid barcodes, which can be concurrently quantified through complementary DNA (cDNA) amplicon sequencing and reflects the transcriptional activity of the associated enhancer within the specific cells where the library has been introduced.

The effective delivery of plasmid libraries containing hundreds to thousands of candidate enhancers to cultured cells has enabled comprehensive and quantitative analysis of how subtle variations in genome sequence correspond to differences in cell-line-specific gene regulation. Numerous studies have used reporter assay techniques to assess the impact of single nucleotide polymorphisms (SNPs) identified in expression quantitative trait loci studies (eQTLs) (Tewhey et al., 2016; Abell et al., 2022), SNPs from genome-wide association studies (GWAS) (Ulirsch et al., 2016; Chaudhri et al., 2020; Myint et al., 2020), and mutations specific to the human lineage (Jagoda et al., 2022; Uebbing et al., 2021). In addition, these high-throughput reporter assays have been modified to investigate gene regulation in cultured neurons (Nguyen et al., 2016; Girskis et al., 2021). However, a critical limitation of applying MPRA technologies to cell cultures, particularly in cultured neurons, is the inherent inability to investigate gene regulation within its natural environment. In the brain, gene regulation displays a high degree of interconnectivity and regional neuron specialization, and is influenced by a plethora of type specific neurons within the network to synchronize their gene regulatory programs (Lawler et al., 2022; Gordon et al., 2013). One approach to overcoming this has been to electroporate dissected newborn retinas *ex vivo* (Zhao et al., 2023; White et al., 2016; Hsiau et al., 2007), but this approach is limited to tissue from newborns and cannot be applied to most tissue types, including most neural tissues. As such, the full complexity of gene regulation cannot capture the transcriptional regulatory network of *in vivo* neural tissue (Lawler et al., 2022; Bagot et al., 2016). Indeed, recent studies demonstrate significant disparities between transcriptional regulatory networks of cell culture models and those present in *in vivo* neural tissues (Kitsis and Leinwand, 1992; Lopes-Ramos et al., 2017; Kaplow et al., 2023).

To overcome the limitations of MPRA technologies applied to cultured cells, MPRAs have been adapted to explore the comprehensive complexity of gene regulation in neural tissues *in vivo*. Several studies have employed *in vivo* MPRAs, utilizing *in utero* electroporation and adeno-associated viruses (AAVs) (Hrvatin et al., 2019; Shen et al., 2016; Lambert et al., 2021; Warren et al., 2022; Mulvey et al., 2021), injection into embryos (Kvon et al., 2020), or stereotaxic injections of AAV which entails drilling a hole through the skull and injecting into the mouse brain (Chan et al., 2023), for MPRA library delivery. These studies demonstrate the capability and sensitivity to measure tissue or cell-type specific enhancers (Blankvoort et al., 2018). However, an inherent challenge lies in effective delivery methods of these MPRA libraries to cells *in vivo*, resulting in a limited ability to transduce the libraries in multiple brain regions. This hinders the comprehensive detection of the impact of genetic variants on brain neural tissue gene regulation (Lambert et al., 2021; Mulvey et al., 2021). Currently, MPRA technologies and the key limitations are extensively reviewed by Degner and colleagues (Degner et al., 2025). In this study, using our sysMPRA technology, we have overcome key challenges associated with stereotaxic injection, pioneering a robust and systemic approach for delivering MPRA enhancer libraries across multiple tissues within a single organism in comparison with the current available MPRA technologies. These advantages enable a comprehensive capture of gene regulation dynamics in non-coding genome regions across the brain and a diverse array of other tissues.

The power of an MPRA experiment is proportional to the number of cells that take up a given library. We chose systemic AAV to maximize transduction *in vivo*. Lentivirus-based methods have proven invaluable for *in vitro* MPRA (Inoue et al., 2017; Gordon et al., 2020) and for organoids (Capauto et al., 2024; Kosicki et al., 2024), but it is not able to cross the blood brain barrier to effectively transduce large amounts of tissue. However, there may be a loss of information due the AAV-based methods not integrating into the genome in contrast to the lentivirus (Inoue et al., 2017). The spread and rate of transduction in the adult mouse brain is not sufficient for a high-throughput approach.

The STARR-Seq assay is a version of the massively parallel reporter assay. The most notable difference is that the enhancer itself serves as the barcode (Arnold et al., 2013). A key feature of the STARR-Seq design that we share is that the enhancer is downstream of the reporter gene, although previous work has found a strong correlation in cases where the synthesized regulatory elements function upstream and downstream (Nguyen et al., 2016). We chose to adapt a version of MPRA with synthesized barcodes for two reasons. First, given that this is new technology, we wanted to reliably measure each enhancer sequence with multiple barcodes, which is not possible if the enhancer itself is the readout. Second, if the synthesized sequences are only different by one nucleotide, the readout may not contain the genetic variant itself without more costly long read sequencing technology.

Here we present, an *in vivo* MPRA technology that compares hundreds of candidate enhancers’ ability to activate transcription across multiple brain regions and tissues within a single animal. We developed an innovative systemic massively parallel reporter assay (sysMPRA) by integrating a custom designed, highly modular plasmid, with a previously described AAV-PHP. eB virus (Chan et al., 2017). This combination enables the efficient delivery of the MPRA library containing our reporter assay to various tissues with high reproducibility within a single animal (Figure 1). The AAV-PHP. eB viral serotype enters the brain by crossing the blood-brain barrier, enabling a quick and minimally invasive intravenous injection method instead of direct injections into brain tissue. This approach allowed us to address the challenges linked to direct injection by delivering the MPRA library across multiple brain regions, while simultaneously facilitating viral delivery to various mouse tissues and therefore, offering a more robust MPRA application. We show effective transduction of the MPRA library into a variety of mouse tissues and confirm its ability to proficiently identify tissue specificity in candidate enhancers, which includes a particular focus on neural and liver tissues. Our MPRA technology enables direct comparisons within a single mouse of enhancer activity between different brain regions and brain versus other tissues. We demonstrate that sysMPRA efficiently detects the effects of synthetic disruptions of candidate transcription factor binding sites, SNPs, and naturally occurring human variants on tissue-specific enhancer activity. Thus, our sysMPRA highlights crucial improvements *in vivo* MPRA technologies and allows the sensitivity to capture the full dynamics of gene regulation of non-coding genome regions across the brain in its natural environment.

## Results

### SysMPRA libraries are successfully delivered to tissues across the mouse

To evaluate the efficiency of delivering our sysMPRA libraries to diverse mouse tissues, we employed a delivery system optimized for enhanced transduction and reproducibility. We designed the sysMPRA plasmid (pAAV-MPRAe) with an Hsp68 minimal promoter, as previously described by Lambert and colleagues (Lambert et al., 2021). This ensures maximum inducibility of transcription without driving high levels at baseline, a crucial feature for assessing library delivery. The candidate enhancer sequences and barcodes (MPRA insert) were cloned downstream of both the minimal promoter and the mCherry reporter. We introduced cloning sites within the plasmid backbone allowing for easy modular change of both promoter (promoter cloning sites) and enhancer sequences (MPRA cloning sites) (Supplementary Figure S1).

**FIGURE 2:**
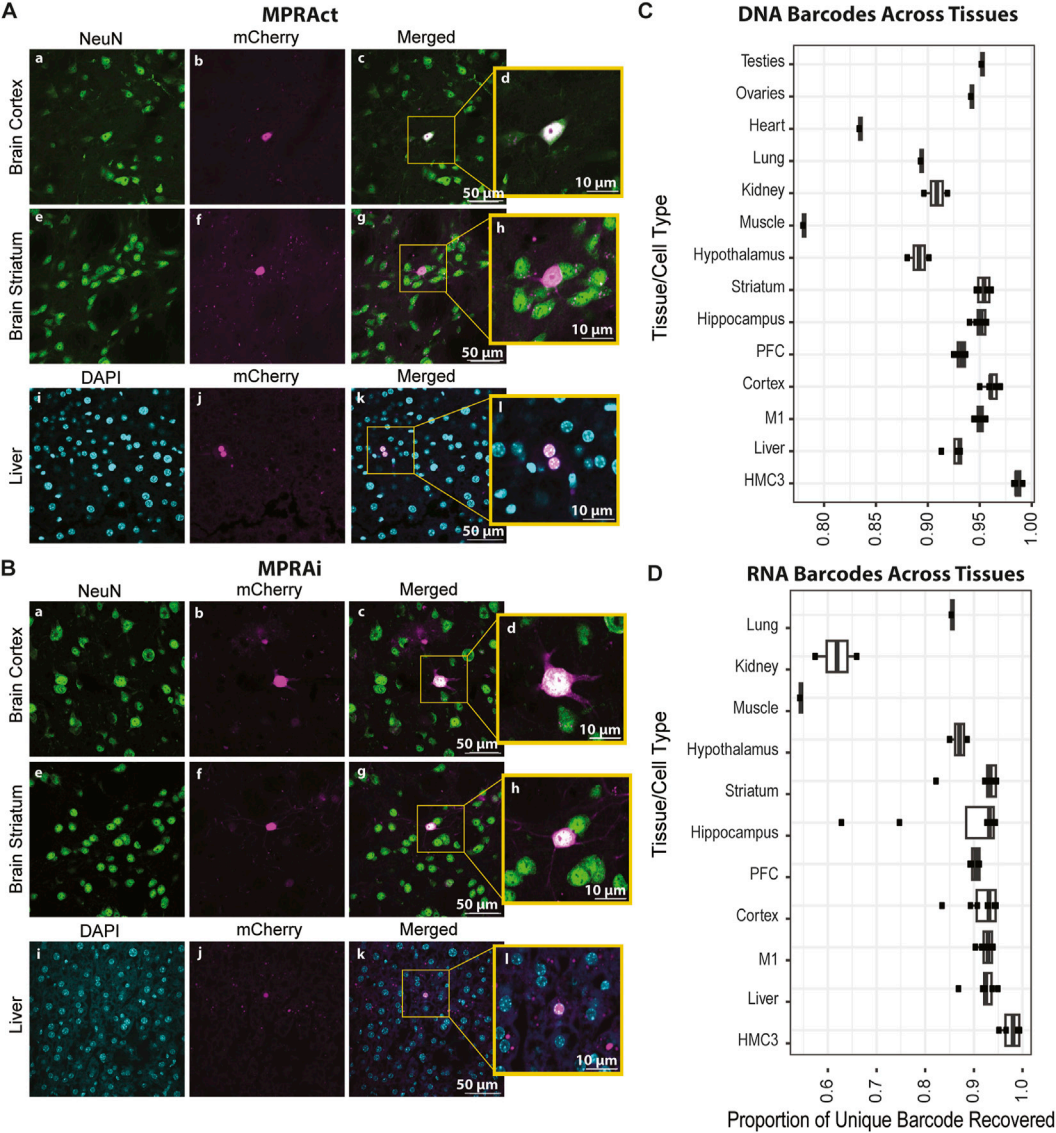
SysMPRA delivers the MPRA libraries across tissues *in vivo*. **(A)** Confocal images of mCherry expression from MPRAct library (cross-tissue positive controls). Shown is mCherry (magenta) compared to NeuN expression (green) in the brain cortex (panels a–d) and brain striatum (panels e–h) from a C57Bl/6J mouse. mCherry (magenta) is also compared to DAPI (blue) expression in the liver (panels i–l) from a C57Bl/6J mouse. **(B)** Confocal images of mCherry expression from MPRAi library (MPRA library of 461 enhancers/variants). Shown is mCherry (magenta) compared to NeuN expression (green) in the brain cortex (panels a–d) and brain striatum (panels e–h) from C57Bl/6J mouse. mCherry (magenta) is also compared to DAPI (blue) expression in the liver (panel i–l) from a C57Bl/6J mouse. **(C)** Plot of unique DNA barcodes present in multiple mouse tissues, which serves as a metric to assess efficiency in MPRA library transduction. **(D)** Plot of unique RNA barcodes present in various mouse tissues, which serves as a metric for assessing the ability of the MPRA library to drive expression of candidate enhancers.

First, we assessed the transduction and expression of our mCherry reporter system by testing 3 cross-tissue positive control enhancer sequences each with a unique barcode (Supplementary Table S1). These sequences were cloned into the sysMPRA plasmid to create a test MPRA preliminary library (MPRAct) which allowed us to provide an easy means to confirm the functionality of our sysMPRA approach. We transduced the MPRAct library into wildtype adult mouse tissue, and we evaluated the transduction and transcription in targeted tissues, including neurons in multiple brain regions, by measuring mCherry fluorescence in tissue sections (Figure 2A). Indeed, our data clearly indicates the effective performance of the MPRAct library and, in turn, validates the sysMPRA approach.

Next, we implemented our designed library of 461 enhancers each paired with 20 unique barcodes (MPRAi) to assess the ability of sysMPRA to relate differences in genome sequences to regulatory differences (Figure 1A; Supplementary Table S2). The relatively large number of barcodes ensures that each enhancer is well-represented, even in the event of dropout at the cloning stage. We speculate that potential dropout rate can likely be induced by the barcode sequences integrating into the mRNA sequence template, thereby potentially impacting mRNA stability and translation directly. To establish a comprehensive library for our study, we designed the MPRAi library with the following components: (1) We included a collection of anticipated positive and negative controls derived from brain, liver, and immune cells, referencing their documented regulatory activity in prior MPRA experiments (Kheradpour et al., 2013; Nguyen et al., 2016). (2) We incorporated 144 candidate enhancers into the MPRAi library that are highly conserved and have mouse cortex H3K27ac chromatin immunoprecipitation sequencing (ChIP-seq) regions near genes implicated in vocal learning as well as their orthologs across species (see Methods). This aims to evaluate the potential of these enhancers to regulate genes associated with vocal learning and to improve the overall signal from mouse brain tissue (mouse brain specific candidate enhancers). (3) We introduced a set of 28 sequences known to bind the transcription factor MEF2C, implicated in transcriptional regulation across multiple brain regions (Harrington et al., 2016; Chen et al., 2016) as well as Alzheimer’s disease (AD) predisposition (Karch and Goate, 2015). This addition enables testing our sysMPRA technology to detect the impact of disrupting transcription factor binding sites. (4) We integrated a set of 27 candidate enhancers containing both the risk and the non-risk alleles of candidate regulatory AD-associated variants from GWAS (Lambert et al., 2013). This inclusion allows for the detection of the impact of SNPs.

The designed MPRAi library was cloned into the plasmid backbone to create the plasmid library (pAAV-MPRAi) (Figure 1B) and subsequently, we delivered the constructed MPRAi library into the brains of mice using retro-orbital injection of AAV-PHP. eB as described previously by our group (Lawler et al., 2022) (Figure 1C). We collected the mouse tissues and performed sectioning on the liver, frontal cortex, and the striatum. Next, we analyzed the mCherry fluorescence of the nuclear-associated reporter relative to NeuN (labels neurons, used to evaluate brain) or DAPI (labels nuclei, used to evaluate liver) levels by using confocal imaging (Figure 2B). Indeed, mCherry fluorescence was detected in liver, brain striatum and brain cortex cells, a good indication of systemic viral transduction of the MPRAi library in multiple tissues (Figure 2B).

Then, we analyzed the MPRAi plasmid library complexity itself to assess possible drop-out effects of the MPRAi library assembly caused by the cloning procedure. This allowed us to ascertain how many of the originally synthesized 20 unique barcodes were still present in the final library. This is an important metric, as it gives means to correct the number of barcodes actually present in the MPRAi library and therefore a more accurate assessment of the viral transduction rates of the sysMPRA technology. We performed plasmid DNA sequencing by using Illumina MiSeq and discovered that the MPRAi plasmid library complexity is 43% of the input library complexity. Thus, our MiSeq data show a 57% drop-out of barcodes introduced by the cloning procedure, but we still have 100% coverage of the candidate enhancers. Despite the unexpected and significant drop-out in the MPRAi library, we reasoned that an adequate number of barcodes per enhancer would be still available if our sysMPRA is functioning efficiently. Our sequence data analysis of the barcodes per enhancer showed a range of 1–18 barcodes, with an average of 8.6 barcodes (Supplementary Figure S2). Over 90% of the enhancers were associated with at least 5 unique barcodes in the final library (Supplementary Figure S2). In the future, adapting the cloning procedure is highly likely to yield a higher barcode library coverage.

Subsequently, we further investigated the viral transduction efficiency as well as the MPRAi library expression across various tissues by measuring the library complexity based on the number of unique barcodes detected at DNA and RNA level. The ability of the sysMPRA to detect transcriptional differences across tissues depends on the complexity of the library that can be transduced into each tissue. Enhancer regulatory activity is highly cell type- and tissue-specific (Jindal and Farley, 2021). Thus, the unique DNA barcodes recovered should be a function of the libraries ability to transduce. The unique RNA barcodes recovered will be a function of the set of designed enhancers to drive barcode expression. To measure transduced library complexity in each tissue, we counted the number of barcodes present in the viral DNA reads (Figure 2C). We found that most samples (excluding heart and muscle) across all tissues contained greater than 89% (ranging from 89% to 96%) of all measured barcodes (Figure 2C). The complexity of the brain regions and other tissues were only slightly less than complexity measured in transfected HMC3 cells (Figure 2C). This demonstrates our ability to efficiently transduce a complex library across a broad set of mouse tissues using AAV-PHP. ebb. The proportion of RNA barcodes recovered dropped, most likely due to poorly expressed candidate enhancers (Figure 2D). As expected, there was a greater drop in RNA barcodes relative to DNA barcodes for tissues outside the brain due to the library construction being heavily brain enhancer-focused.

Next, we cataloged the transduction within our MPRAi library by measuring the levels of unique barcodes across the various mouse tissues. Similarly, as before, we injected (retro-orbital) the library into 10 mice and collected the samples from multiple tissues (Supplementary Figure S3). Furthermore, we introduced the MPRAi library into the microglia-like HMC3 cell line (Dello et al., 2018), aiming to compare our sysMPRA *in vivo* technology with cell culture technologies. This approach simultaneously enabled us to investigate the potential function of MEF2 binding sites and AD-associated genetic variants, as previous studies have implicated both factors to microglia (Deczkowska et al., 2017; Gjoneska et al., 2015a). We employed a custom program, arrayProc.2.1.1. py, to analyze unique barcodes in each sequenced sample, enabling the quantification of barcode reads at the DNA level (see Supplemental Methods, computational analysis). Then, we refined the sequence data, retaining only high-quality barcode reads, which we defined as reads that matched with both the designed restriction enzyme site within the viral plasmid and the adjacent bases of the barcode. Our results demonstrate the identification of 3,983 high-quality unique barcodes across multiple tissues (Supplementary Table S3). This corresponds to an overall MPRAi library transduction rate of 95.6%, calculated as 3,983 (identified barcode sequences) divided by the total MPRAi library input barcodes 4,149 (461 × 9 barcodes). In other words, the MPRAi library drop-out was only 4.4%, meaning that we were able to detect, on average, the majority (8.6) of the 9 barcodes for each candidate enhancer.

Next, we evaluated the Spearman Rho (ρ) correlation of the plasmid DNA barcode measurements from each pair of samples. The ρ coefficients ranged from ρ = 0.737 to ρ = 0.991, with a median of ρ = 0.951 **(** Supplementary Figure S4A; Supplementary Table S3). These findings clearly demonstrate that the identified barcodes of each sample are highly correlated across the various tissues. To get a clear overview of the number of detected barcodes within the various tissues, we displayed each tissue type (sample) and calculated the proportion of high-quality unique barcodes detected in the viral DNA ranging from no barcodes detected (0.0) to all barcodes detected (1.00, corresponding to 3,983 barcodes) (Supplementary Figure S4B). We show that each tissue sample and the HMC3 cell line has a high proportion of high quality unique barcodes detected, ranging from 0.82 (muscle tissue 10_2) to 0.99 (HMC3_C2), with an average of 0.94 ± 0.035. Thus, at minimum, we were able to detect 0.82 × 3,983 = 3,266 unique barcodes and at maximum 3,944 barcodes, a strong indication of widespread transduction (Supplementary Figure S4B). Collectively, these results unequivocally showcase the widespread transduction of our MPRAi library throughout all mouse tissues. Thus, our sysMPRA technology demonstrates its efficacy in facilitating robust *in vivo* transduction of the library.

### SysMPRA measures the tissue-specificity of candidate enhancers

To investigate the capability of sysMPRA to efficiently detect tissue-specificity of the candidate enhancers, we measured the RNA barcode expression across all the different tissues. This would allow us a direct comparison of the candidate enhancer activity across the various tissues. Leveraging a wealth of expertise accumulated over nearly a decade in studying neurobiology and non-coding regions within neural tissues, our special focus was directed to candidate enhancers in brain tissues. Additionally, we incorporated candidate enhancers for liver and immune cells to explore diverse tissues, evaluating the broad applicability of sysMPRA in detecting potential enhancer activity across animal tissues. This aimed to assess the robust functionality of the sysMPRA technology. It is crucial to recognize that the chosen panels of candidate enhancers tailored for the brain, liver, and immune-like cells (HMC3) are tissue-specific. This implies that the potential enhancer activity of these candidates will be most pronounced where the gene regulatory machinery is prevalent in the respective tissues. In simpler terms, enhancers designed specifically for the brain are likely to be active in the brain tissues, with minimal or no activity anticipated in the liver, immune cells or other tissues and *vice versa*.

**FIGURE 3:**
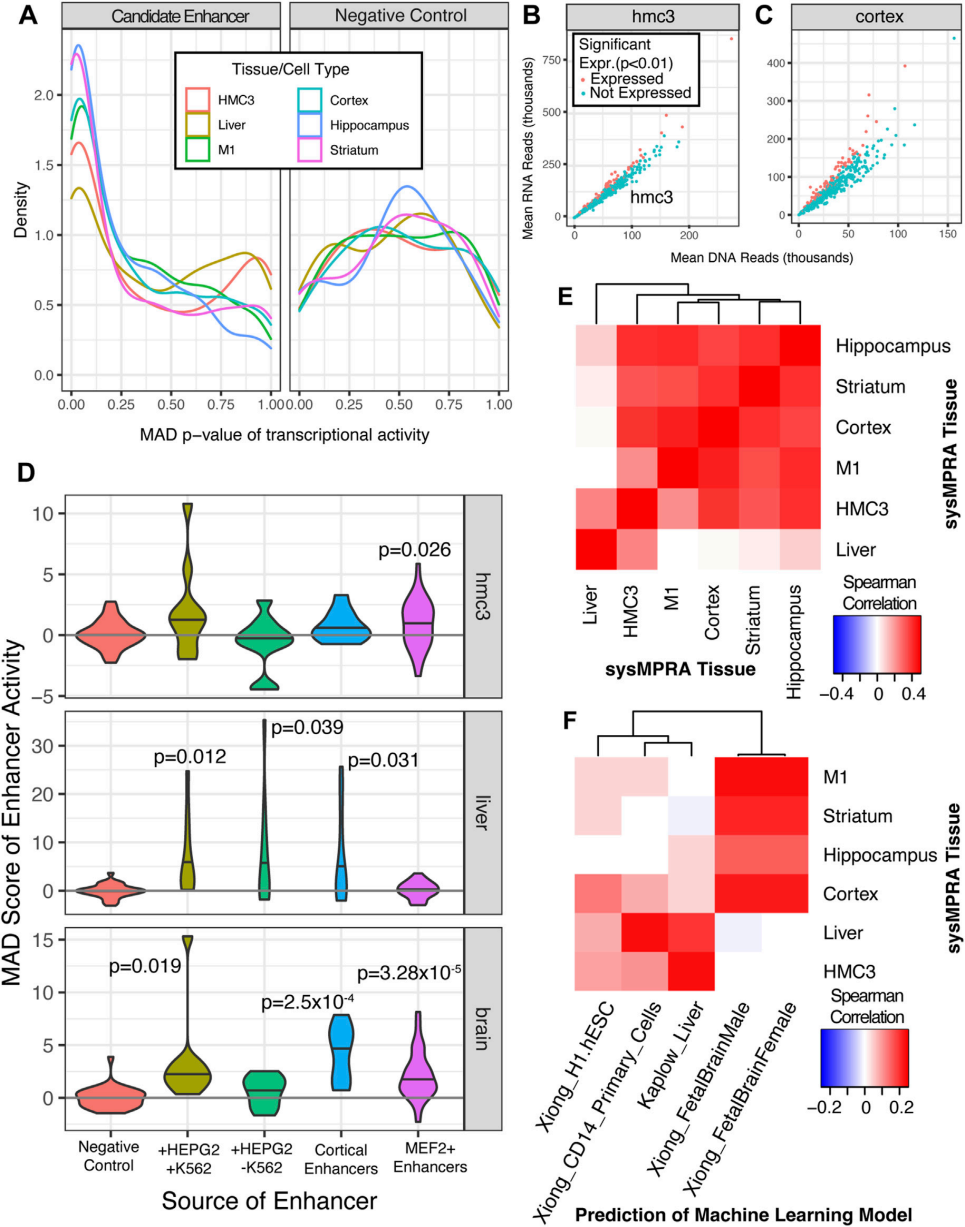
SysMPRA captures tissue-specific signatures of gene regulation *in vivo*. **(A)** The frequency of p-values is displayed using a density plot across all candidate enhancers and positive controls in MPRAi library (left) relative to the negative controls (right). The ratio of RNA reads to DNA reads, which roughly corresponds to transcriptional activity, is plotted for **(B)** HMC3 cultured cells and for **(C)** cortical tissue. The mean across all samples for that tissue is used **(D)** The MAD score is displayed as a violin plot for the positive and negative control enhancers gleaned from other MPRA experiments in HMC3 cells, liver and brain tissues as well as candidate enhancers with MEF2C binding sites. The p-values are based on a t-test of the mean value across each sample. **(E)** Spearman’s rho is calculated across the estimated transcription rate, alpha, of all enhancers for each pairwise tissue comparison. **(F)** Spearman’s rho is calculated between the estimated transcription rate, alpha, of all enhancers and the prediction of open chromatin levels calculated by convolutional neural network models.

To measure the barcode RNA expression, we extracted RNA from the various mouse tissues and performed RNA sequencing on the samples that passed our rigorous quality control with the Illumina NovaSeq (Figure 1D; Supplementary Figure S3). The barcode RNA expression levels were assessed by the amount of RNA barcodes detected in the tissue (RNA counts) of interest as it is a direct measurement of mRNA level expression. First, we used the RNA barcode counts (Supplementary Table S5) relative to the DNA barcode counts (Supplementary Table S4) to estimate the activity of all candidate enhancers with MPRAnalyze (Ashuach et al., 2019). We found that the tissue-specific candidate enhancers, including the likely positive control enhancers for these tissues (HMC3, liver, M1, cortex, hippocampus and striatum), had a strong tendency to be expressed relative to the negative control sequences (Figure 3A). The p-values of the MAD score for the candidate enhancers is extremely low, nearing zero, strongly suggesting high transcriptional activity as compared to the negative controls, which exhibit a shift in MAD score peak p-values toward higher values (between 0.35 and 0.80) (Figure 3A). Moreover, we directly compared the cortical candidate enhancers to the positive and negative controls, demonstrating that these enhancers are likely to drive substantial activity in the cortex (Supplementary Figure S5). Notably, two-thirds of positive controls and half of our candidate enhancers with MEF2 motifs activated transcription in the cortex. Compared to the positive control, the candidate cortical and MEF2 enhancers have similar distribution of MAD scores and have lower median MAD scores (Supplementary Figure S5A). Similarly, the proportion of candidate cortical and MEF2 enhancers were disproportionately enriched to have significant transcriptional activity (P < 8.210^-8, Supplementary Figure S5B). These findings align with expectations for cortical candidate enhancers in comparison to the positive controls. Subsequently, we investigated the candidate enhancers for activity in cultured HMC3 cells (Figure 3B) and for the brain tissues **(** Figure 3C) by ratioing the DNA versus RNA reads, as it is a good proxy for the transcription activity of the enhancers. We show that, among the panel of candidate enhancers (461), numerous exhibit transcriptional activity in cultured HMC3 cells (Figure 3B) and in the brain tissues (cortical tissue) (Figure 3C). This is demonstrated by the significant detection of mRNA levels (RNA barcodes) expressed for several enhancers (red dots, p < 0.01) in both the HMC3 cells and the brain tissues **(** Figures 3B, C). These findings also reveal that the most of our active candidate enhancers are predominantly identified in the brain and indeed align strongly with our expectations, considering the majority of candidate enhancers are primarily designed for brain tissues. Furthermore, when we compared the quality control metrics between the *in vivo* and HMC3 version of the experiments (Supplementary Table S3), we found that the data quality and signal distributions were similar. For example, the RNA:DNA ratios from the HMC3 cells (Figure 3B) showed comparable spread to the RNA:DNA ratios from brain tissue, like cortex (Figure 3C), a strong indication that the quality control metrics between the *in vivo* and *in vitro* experiments are consistent.

Next, we dissected the panel of positive control candidate enhancers in the MPRAi library in more depth by analyzing the MAD score of enhancer activity in the various tissues (HMC3, liver and brain) relative to the negative control candidate enhancers. This allowed us to evaluate our sysMPRA experimental approach to identify tissue-specific differences in enhancer activity in living mice. We tested the set of candidate enhancers active in both HEPG2 (liver-like) and K562 (immune) cells (Kheradpour et al., 2013), which showed a nominal trend toward expression in HMC3 cells (one-sided t-test p = 0.078), the liver (one-sided t-test p = 0.012), and the brain tissues (one-sided t-test p = 0.019) (Figure 3D). As expected, HEPG2-specific enhancers (liver) tended to be transcribed in only the liver (one-sided t-test p = 0.039). The set of control enhancers for cortical tissue (brain tissue) and MEF2+ enhancers (brain tissue) revealed the highest enhancer regulatory activity in the brain (one-sided t-test p = 0.00025 and p = 0.0000328, respectively) (Figure 3D). These results clearly demonstrate that the overall expression patterns of control candidate enhancers in the MPRAi library align with our expectations. Consequently, it proves that our sysMPRA technology identifies tissue-specific differences in enhancer activity *in vivo*, at least for the designed positive controls, and validates our experimental approach.

Subsequently, we evaluated the expression patterns across all candidate enhancers by calculating the Spearman’s Rho correlation between various sysMPRA tissues. We found a statistically significant correlation between enhancer activity across different brain tissues (Spearman Rho ρ = 0.348 to ρ = 0.433) (Figure 3E) and little correlation between brain *versus* liver enhancer activity (Spearman Rho ρ = 0.0018 to ρ = 0.0971) (Figure 3E). Indeed, this data aligns with our expectations, given that the vast majority of the candidate enhancers in our MPRAi library are designed for brain tissues, with only a few targeting the liver tissue. Furthermore, we included the microglia-like cell line (HMC3) in our calculations and showed significant correlations between enhancer activity across brain tissues (Spearman Rho ρ = 0.225 to ρ = 0.407) (Figure 3E). This can be explained by the fact that microglia cells are specialized immune cells residing in the central nervous system that play critical roles in the brain during the development, homeostasis, and pathologies and therefore can overlap with the MPRAi library candidate enhancer activities (Figure 3E). Interestingly, our data suggest that the microglia-like cell line has the strongest correlation with the hippocampus and cortex and indicates a moderate correlation with the MPRAi library candidate enhancer activity in the liver (Spearman Rho ρ = 0.225) (Figure 3E). This may be because many of our candidate enhancers are bound by the transcription factor MEF2C, which is known to play an important role in microglia (Deczkowska et al., 2017) Overall, our findings suggest that the estimated enhancer activity levels can reliably gauge regulatory activity across various tissues by our sysMPRA technology.

To verify the relevance of the enhancer activity we measured (Figure 3E) to the tissue-specific regulatory code, we compared the activity measured across all enhancers to machine learning model predictions of open chromatin (Kaplow et al., 2022; Zhou and Troyanskaya, 2015). Open chromatin prediction models are known to be correlated with enhancer activity (Roadmap et al., 2015) (Supplementary Table S6). To ensure that our MPRA could quantify tissue-specific regulatory activity, we assessed how well a model trained in a cell type for which our candidate enhancers were not designed to be active could predict MPRA regulatory activity; poor performance would indicate that the MPRA’s quantifications were tissue-specific. We found there was a weak but mostly positive correlation with the machine learning models trained in human embryonic stem cells Spearman Rho ρ = −0.001–0.076.

We then assessed the predictions of machine learning models trained on brain tissue open chromatin and observed significant correlations with sysMPRA measured enhancer activity in brain tissue (Spearman Rho ranching from ρ = 0.121 to ρ = 0.183 with p-values from 9.39 × 10^−3^ to 7.90 × 10^−5^). No correlation was found in liver tissue (Spearman Rho = −0.0117; p = 3.7 × 10^−3^) (Figure 3F). Similarly, the predictions from the machine learning models trained on liver open chromatin were significantly correlated with sysMPRA enhancer activity in liver (Spearman Rho = 0.158; p = 6.68 × 10^−4^) but not brain (Spearman Rho from −0.0112 to 0.0341 with p-value 0.466–0.838) (Figure 3F). Given that HMC3 cells model microglia, we tested whether machine learning models for a similar cell type, CD14^+^ macrophages (London et al., 2013), were able to capture HMC3 regulatory activity. We found a weak correlation between HMC3 regulatory activity predicted CD14^+^ monocyte open chromatin (Spearman Rho = 0.084; p = 0.07). The correlation was higher with liver, a tissue known to contain a large proportion of CD14^+^ macrophages (Spearman Rho = 0.184; p = 0.0007) (Dixon et al., 2013).

Finally, we conducted a thorough analysis of the RNA barcode expression in all sysMPRA transduced tissues from the mice and HMC3 cells (Figure 1D; Supplementary Table S5). This aimed to gain comprehensive insights of the activity of candidate enhancers in a wide range of tissues. Similarly, as described above, we evaluated the Spearman Rho (ρ) correlation and show a wide variety in the Spearman Rho correlation ranging from (ρ) = 0.366 to (ρ) 0.995 with a median of (ρ) = 0.629 (Supplementary Figure S4C; Supplementary Table S3). Our results consistently demonstrate a strong correlation between various brain tissues (M1, cortex, striatum, hippocampus, hypothalamus) and HMC3 cells, but low correlation in liver tissue (Supplementary Figure S4C; distinct colored red squares). This strongly suggests that the designed candidate enhancers for brain tissues exhibit a robust tendency to be active, leading to the expression of RNA barcodes. These findings match with MAD scores p-values, including positive controls for enhancer activity in liver (HEPG2), immune cells (K562), and cortical tissue as well as candidate enhancers with MEF2 transcription factor binding sites; they also align with Spearman Rho correlations for brain tissues, HMC3 cells, and liver tissue (Figure 3). As such, sysMPRA performs effectively in identifying tissue-specific transcriptional regulation of enhancers tailored for the specific tissues (brain, HMC3, and liver). The MPRAi library’s candidate enhancers, which are not designed for tissues like kidney and muscle, show consistently lower Spearman Rho correlations (Supplementary Figure S4C; different shades of blue-colored squares). These results suggest that the majority of these candidate enhancers are unlikely to exhibit significant activity in non-target tissues. However, there are a few exceptions. For example, some MPRAi library candidate enhancers show low activity in lung tissue relative to HMC3, cortex, and hippocampus and others have low enhancer activity in kidney tissue relative to HMC3 (Supplementary Figure S4C; lighter shades of red-colored squares). This indicates that a few MPRAi candidate enhancers might have some activity in lung and kidney tissue.

In addition, our results demonstrate that the RNA barcodes are less reproducible across samples (Supplementary Figure S4C; median of (ρ) = 0.629) than the DNA barcodes (Supplementary Figure S4A; median of ρ = 0.951). It is important to realize that these two entities represent completely different dynamics. DNA barcodes represent the viral transduction of the MPRAi library, indicating its ability to transduce into the various tissues *in vivo*. Meanwhile, RNA barcodes directly correlate with the gene regulatory capacity of the MPRAi library’s candidate enhancers in various tissues. In other words, the expression of RNA barcode is directly influenced by the activity of candidate enhancers in the tissues. Thus, detecting RNA barcodes necessitates some level of enhancer activity, while the detection of DNA barcodes is completely unrelated. For this reason, it is not surprising that RNA barcode detection declines in reproducibility across the tissue samples. Indeed, the highest proportion of unique detected barcodes at RNA levels are demonstrated for brain, HMC3, and liver samples, aligning well with the tailored design of the MPRAi library candidate enhancers for these tissues (Supplementary Figure S4D). Our findings also reveal that most other tissues (muscle, kidney, lung) had a significant proportion of unique RNA barcodes detected with a range of 0.5–0.85 for muscle and lung tissue, respectively (Supplementary Figure S4D). This indicates a significant recovery of 50%–85% of the RNA barcodes from the MPRAi library input in these tissues.

In summary, our data strongly indicates that sysMPRA is capable of evaluating estimated enhancer activity *in vivo*, demonstrating its reliability in measuring tissue-specificity regulatory activity of candidate enhancers across different tissues.

### SysMPRA detects enhancer disruptions from transcription factor binding sites and SNPs

Current *in vivo* MPRA technology lacks the sensitivity to discern subtle activity differences arising from disruptions in individual transcription factor binding sites or SNPs. This limitation hampers its ability to fully capture the dynamic intricacies of gene regulation in non-coding genome regions within brain neural networks in their natural environment. Therefore, if sysMPRA could overcome this crucial limitation, it would allow researchers to study non-coding regions within the natural environment of brain neural networks. To evaluate whether sysMPRA is sensitive enough to detect subtle activity differences arising from disruptions in individual transcription factor binding sites or SNPs, we devised two strategies: (1) examining the influence of disruptions in transcription factor binding site motifs on enhancer activity *in vivo*, and (2) assessing the impact of disease-related SNPs on candidate enhancers and their effects on regulatory activity *in vivo*.

**FIGURE 4:**
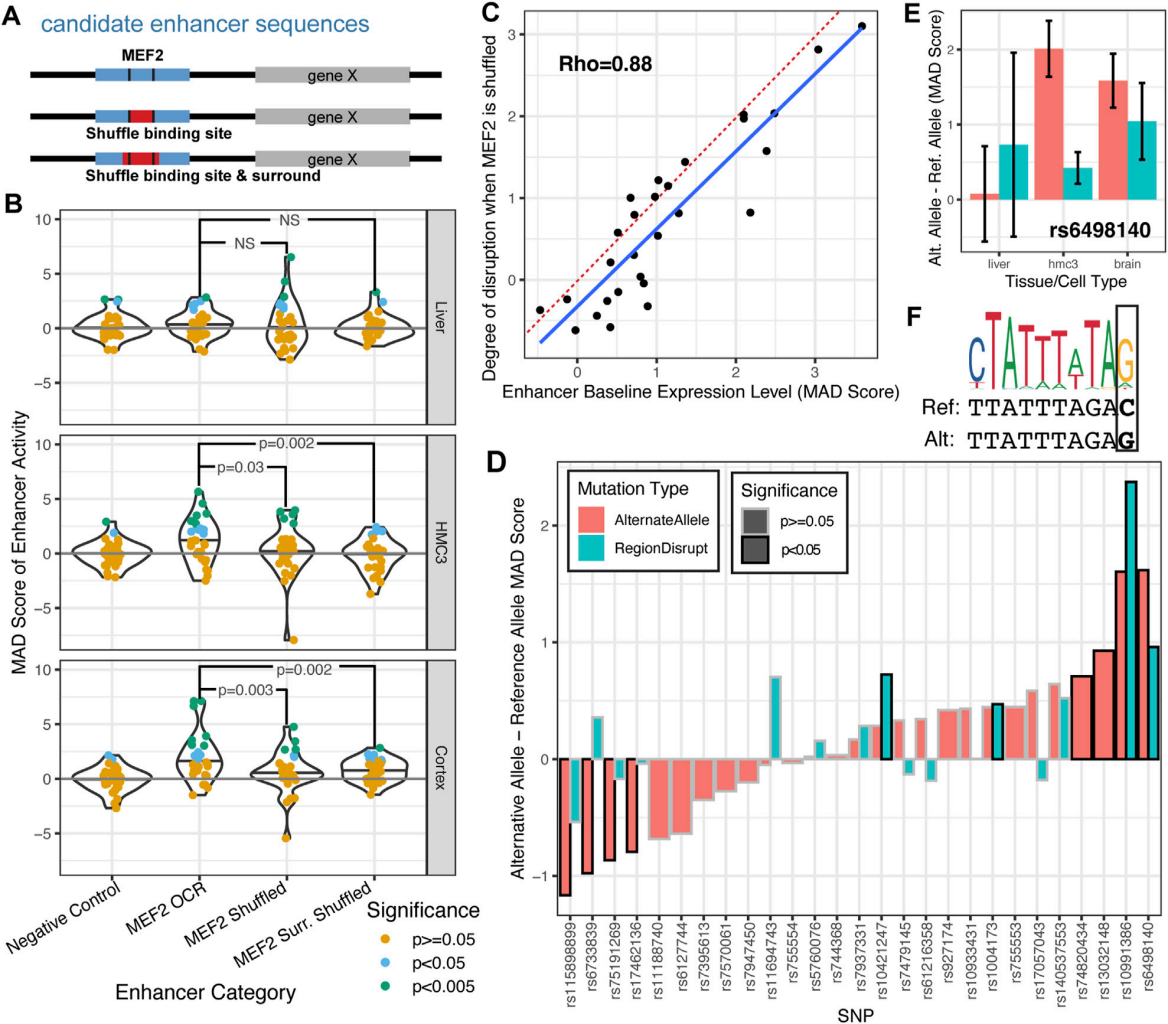
SysMPRA detects enhancer differences due to MEF2C binding site disruption and candidate Alzheimer’s disease SNPs. **(A)** The experimental design of how MEF2C is systematically disrupted at candidate enhancers with binding sites. **(B)** The MAD score of enhancer activity is compared between negative control enhancers and different versions of MEF2C candidate enhancers. Each candidate enhancer is colored based on its nominal significance of transcription relative to the population of negative controls (MAD p-value). **(C)** The MAD score of baseline enhancer expression is compared to the difference between the baseline enhancer expression and the average expression across the two instances of MEF2C shuffling. The degree of disruption is the fold difference calculated using a paired t-test comparing the MAD score of the OCR with the MEF2 motif to the mean MAD score of the OCRs with the shuffled MEF2 motif. The dotted red line shows y = x, while the blue line is fit through linear regression. **(D)** The MAD score of the enhancer activity from the reference allele is compared to the alternate allele (red) and the sequence with a shuffled local transcription factor binding site (blue, “RegionDisrupt”) across brain tissues. **(E)** For one particular SNP, rs6498140 we further stratify by specific tissues. Error bars represent 95% confidence intervals. **(F)** The motif logo for a discovered MEF2 transcription factor binding site is visualized above the reference and alternative allele for rs6498140 and shows a point mutation for C::G.

First, we assessed how disruption in transcription factor binding site motif MEF2 disrupts enhancer activity *in vivo*. As part of the MPRAi library, we designed a set of 28 candidate enhancer sequences based on binding the MEF2 transcription factor in the mouse cortex (see methods). Additionally, we created versions of each enhancer where the transcription factor binding site MEF2 itself was shuffled as well as a version where this motif together with the surrounding 5 nucleotides were shuffled (Figure 4A). We found that the non-disrupted MEF2 motif-containing enhancers had the strongest activity in brain tissue, some activity in HMC3 cells, and no activity in liver tissue (Figure 4B, MEF2 candidate enhancer (MEF2 OCR), panels cortex, HMC3 and liver). This aligns seamlessly with MEF2C’s function in both the brain and microglia (Harrington et al., 2016; Deczkowska et al., 2017; Telese et al., 2015) and by its absence in the liver (Baldarelli et al., 2021). Furthermore, our results on the MAD-scores of enhancer activity MEF2+ as described in this study confirm these results (Figure 3D; MEF2+ enhancers).

Consistent with these observations, we show a significant reduction in enhancer activity in cortical tissue and HMC3 cells upon disruption of MEF2 motifs (Figure 4B). The MAD-scores for the MEF2 candidate enhancers with MEF2 motifs shuffled are significantly lower than those for the original candidate enhancers with MEF2 motifs (Figure 4B; bottom and middle panel; one-tailed t-tests; p-value = 0.003 and p = 0.03, respectively). Additionally, the candidate enhancers with the MEF2 motifs and surrounding sequences shuffled have even lower enhancer activity (Figure 4B; bottom and middle panel; one-tailed t-tests; p-value = 0.002). However, disrupting MEF2 motifs in liver tissue does not result in a significant decline in enhancer activity (Figure 4B; top panel, p > 0.1). Importantly, the extent to which enhancer activity was disrupted by shuffling the MEF2C binding site was strongly correlated with the original baseline expression of the enhancer (Figure 4C; Supplementary Table S7; Rho = 0.88; p = 9.3 × 10^−7^). We found that 7/8 enhancers with a MAD score of >1.3 were significantly disrupted (FDR-adjusted paired t-test p value <0.05) while 0/20 enhancers with MAD score <1.3 were significant. This finding strongly implies that instances where disrupting the MEF2C transcription factor binding site has no impact are situations where the enhancer is inherently inactive in the assay, rather than indicating that the MEF2C binding site is unimportant for enhancer activity.

Next, we investigated the impact of candidate Alzheimer’s Disease (AD) GWAS-derived SNPs on candidate enhancers and their effects on regulatory activity *in vivo* (Figure 4). We utilized our sysMPRA technology to measure the impact of these AD-related SNPs and identified eight SNPs for which the risk and non-risk alleles showed significant (p < 0.05) and divergent activity across brain tissue (Figure 4D; Supplementary Table S8). This highlights that our sysMPRA technology is able to detect subtle enhancer activity differences arising from SNPs in these candidate enhancers (Figure 4D). Two of the eight candidate SNPs (rs6498140 and rs10991386) disrupt a MEF2 motif and for these SNPs, the disrupted motif sequence also displayed a difference from the reference allele (Figure 4D; far right, light blue bars, RegionDisrupt, p < 0.05) with the strongest effect seen for rs6498140 (Figure 4). This candidate AD-associated SNP rs6498140 is proximal to the gene *CLEC16A*, a gene implicated in AD that is a master regulator of autoimmunity and neurodegeneration (Pandey et al., 2023). In comparison to the reference allele (Figure 4E, MAD-score, light blue bars), the alternate allele has the highest regulatory activity in both brain tissue and in HMC3, but not liver (Figure 4E, MAD-score, red bars). This allele creates a MEF2 transcription factor binding site motif in the enhancer by the mutation C (Ref) to G (Alt) (Figure 4F). These findings are consistent with members of the MEF2 transcription factor family demonstrating active transcription in both brain and microglia (Deczkowska et al., 2017; Mitchell et al., 2018). Importantly, rs6498140 displays GTEx eQTL associations in several tissues, notably in the frontal cortex, where the alternate allele correlates with higher expression of *CLEC16A* (Consortium et al., 2013). These findings strongly suggest sysMPRA’s capability to perceive subtle activity differences stemming from SNPs and thereby demonstrating outstanding sensitivity. This not only validates previous studies but also broadens our understanding of genetic variation and gene regulation in Alzheimer’s Disease (AD) within relevant *in vivo* tissues.

Our data showcase that the sysMPRA technology possesses the sensitivity to detect even the most subtle activity differences resulting from disruptions in individual transcription factor binding sites (MEF2) or AD-related SNPs. The technological advantages of sysMPRA not only surpass current limitations in the field but also represent a significant step forward towards studying the dynamic complexities of gene regulation in non-coding genome regions within brain neural networks *in vivo*.

## Discussion

In this study, we present sysMPRA, a technology using systemic intravenous AAV viral delivery to distribute the MPRA library across multiple tissues *in vivo*. We reveal sysMPRA’s robust delivery of the MPRA library, achieving a transduction rate approaching, on average, 96% across diverse animal tissues. This enabled us to effectively show tissue-specific regulatory impacts from candidate enhancers, while also demonstrating that sysMPRA displays the essential sensitivity required to unveil regulatory effects arising from mutations in transcription factor binding sites (MEF2C) and single point mutations (SNPs) associated with both disease phenotype and gene expression.

While MPRA technology has been instrumental in linking genome sequence to regulatory function, thus far it has been used primarily in cell culture. The widespread implementation of MPRA *in vivo* has been restricted due to ongoing significant challenges. These challenges include insufficient viral library delivery as well as limited library quantification across tissues, irregular viral transduction and injection site induced inflammation disrupting gene expression programs. By systemic MPRA library delivery, our sysMPRA technology addresses the current limitations of existing MPRA technologies. We demonstrate effective library delivery and quantification identifying that, on average, 95.6% of the input library is represented by unique DNA barcodes across various tissues (Figure 1D; Supplementary Figure S4B), including a high Spearman correlation (median 0.951) of the DNA barcodes across these tissues (Supplementary Figure S4A). These findings significantly exceed the 66.0%–93.1% range observed in current AAV *in vivo* MPRA studies in terms of the breadth of the delivery across different tissues (Hrvatin et al., 2019; Shen et al., 2016; Lambert et al., 2021; Chan et al., 2023; Cao et al., 2023). In addition, our technology enables us to deliver MPRAs into adult primary tissue, allowing us to evaluate activity of candidate enhancers that may not be active in embryos or newborns, for which some previous MPRA technologies were designed (Shen et al., 2016; Zhao et al., 2023; Lambert et al., 2021; Kvon et al., 2020; White et al., 2013). Furthermore, systemic delivery via retro-orbital injection significantly reduces local inflammation caused by the stereotaxic injection approach used in a previous study (Chan et al., 2023), especially in the brain tissues, and allows for a cleaner viral delivery of the MPRA library without risking gene regulatory processes to be influenced by inflammation. This also enables the study of disease processes of non-coding genomic regions with inflammatory components (Seney et al., 2021; Wyss-Coray and Rogers, 2012). In addition, the AAV serotype used in our sysMPRA crosses the blood brain barrier effectively and therefore prevents high concentration of virus at the injection site, eliminating irregular virus transduction associated with direct injection. Indeed, we observed similar viral transduction in the brain tissues (cortex, striatum, hippocampus, M1) as evidenced by similar levels of unique DNA barcodes detected across these tissues. Moreover, systemic delivery increases the throughput of delivering MPRA library to multiple tissues of interest in the same experimental animal with one brief, minimally invasive procedure, a notable step forward in MPRA technology.

Our findings demonstrate that sysMPRA can be a valuable tool for dissecting regulatory activity of candidate enhancers in a tissue-specific manner. We discovered hundreds of novel candidate enhancers that regulate activity in multiple tissues throughout the animal, aiding in the understanding of the regulatory function of these non-coding genomic regions *in vivo*. Noteworthy, our study primarily focuses on candidate enhancers with a majority targeting the brain and some in the liver tissues, as well as microglia-like HMC3 cells. In the brain and HMC3 cells, the RNA barcode expression is highly correlated (Supplementary Figure S4C, red colored quadrant right upper), and many enhancers are highly active with similar MAD score p-values (Figures 3A–C). This may be because several of the candidate enhancers we tested have motifs for MEF2 transcription factors, which are known to play important roles in both neurons and microglia (Harrington et al., 2016; Deczkowska et al., 2017). Also, the positive controls for both brain (cortical enhancers) and liver (HEPG2 enhancers), as well as candidate enhancers with MEF2 transcription factor motifs had good MAD-score p-values, indicating high enhancer activity in the tissues in which these enhancers are expected to be active (Figure 3D). Our findings solidify sysMPRA’s efficacy in identifying tissue-specific transcriptional regulation of candidate enhancers designed for these tissues. Crucially, this emphasizes sysMPRA’s potential as a pivotal tool for the research community, facilitating comprehensive studies on the regulatory functional roles of non-coding genomic regions in diverse tissues *in vivo*, aligning with their specific research needs.

Our results show that sysMPRA effectively exposes functional differences in regulatory activity caused by mutations and shuffling of transcription factor binding motifs. By analyzing the disruption of MEF2C transcription factor binding sites, our approach dissects the regulatory activity impact in dozens of candidate enhancers known to be active in the brain and microglia (Figure 4). This provides a robust tool for identifying and assessing the specific role of these sequence features and their effects on regulator activity *in vivo*. Moreover, we demonstrated the impact of disrupting individual candidate AD-associated variants on the activity of 28 enhancers. Within this set, we identified 8 risk allele SNPs significantly influencing enhancer activity, with the most pronounced effect observed for the SNP rs6498140 (Figure 4). The causality by allelic replacement of this alternate allele aligns with increased expression of *CLEC16A* in the frontal cortex (Consortium et al., 2013) and is associated with AD. Our findings highlight that sysMPRA can provide comprehensive insights into sequence disruptions, including into disease-associated SNPs, that might contribute to transcriptional disease pathology, thereby enabling us to advance our understanding of enhancer biology and the pathophysiology of neurological and other tissue-specific disorders.

Our sysMPRA *in vivo* technology provides an effective means to detect tissue and allele-specific effects on regulatory activity of candidate enhancers. However, there are limitations in sysMPRA. In contrast to the hundreds of enhancers we profiled, current cell culture reporter assays can provide high quantitative, cell line-specific information across thousands of enhancers (Kheradpour et al., 2013; Jagoda et al., 2022; Ernst et al., 2016). Efficient viral transduction of various tissues across the animal using PHP.eB serotype can be a current limitation of our technology as we measure significantly lower viral transduction in muscle and heart tissue (DNA barcode recovery). Hence, customizing sysMPRA using diverse AAV serotypes and cell-type-specific candidate enhancers is crucial for more efficient targeting of these specific tissues. Additionally, any AAV tropism inherent in PHP. eB serotype or another systemic delivery system can impact how the enhancer functions in the targeted tissues (Brown et al., 2021). For example, gene regulatory programs active in brain microglia are not likely to be captured in our sysMPRA assay due to the bias that PHP. eB has for neurons and other glial cells (Chan et al., 2017). Choosing different viral packaging serotypes might address this limitation. Finally, we observed some variability in RNA barcode drop-out across replicates. This is likely caused by several factors including transduction efficiency variability across animals, amplicon PCR stochasticity and sensitivity issues with recapturing RNA barcode amplicons with low viral representation. We believe it is highly probable that stochastic alterations in the number and the percentage of the transduced cell population between the animal replicates could significantly affect the apparent activity of RNA barcode expression and therefore can produce variability in the candidate enhancer activity between some replicates within the sysMPRA assay. We also note that, for many candidate enhancers, we lost the majority of corresponding barcodes during the cloning procedure. Given the wide variety of tissues we target in the animal simultaneously, optimizing the AAV viral titer concentration, utilizing different serotypes with targeted infectivity for specific tissues of interest, and addressing the high drop-out issues in library complexity during the cloning procedure can significantly decrease the variability across some replicates with sysMPRA.

AAV viral delivery of MPRA libraries remains in its infancy. As advancements in AAV MPRA technology continue, we anticipate increased flexibility for sysMPRA. Exciting progress in the field includes the design of novel AAV variants, enabling the targeting of sysMPRA libraries to specific cell subtypes (Bryant et al., 2021; Öztürk et al., 2021) and extending to non-human primate tissues (Goertsen et al., 2022). As transduction efficiency progresses, sysMPRA holds the potential to be coupled with methodologies for isolating individual cell types (Lawler et al., 2020; Mo et al., 2016) or even facilitating single-cell profiling (Hrvatin et al., 2019; Zhao et al., 2023).

GWAS and whole-genome sequencing studies are identifying an increasing number of candidate regulatory variants underlying the predisposition to complex traits. Fine-mapping and functional characterization of those variants is an important step in connecting genetic predisposition to disease pathophysiology. Although *in vitro* high-throughput reporter assays offer an avenue for high-throughput functional characterization in human cells, there are genetic variants that exert their effects in a specific cell type or tissue environment that cannot be addressed with *in vitro* assays. Complementary to *in vitro* MPRA technologies, sysMPRA provides a platform for high-throughput functional characterization across various tissues within a live organism. This enables tissue-specific regulatory effects to be measured in animal models of disease, including potentially non-traditional model organisms.

## Materials and methods

### Array design

#### Cross-tissue (ct) positive controls

The goal of sysMPRA is to produce robust delivery of plasmid expression vectors to a tissue of interest using AAV. Since transduction and transcription of episomal plasmid DNA are both properties of the virus serotype and regulatory element, we diversified our potential to identify tissues where sysMPRA would work by using 3 viral promoters and enhancers. We selected the 72 bp SV40 enhancer element; the 245 bp SV40 promoter, which includes 1 copy of the 72 bp enhancer (Benoist and Chambon, 1981); and the 305 bp CMV promoter (Thomsen et al., 1984) for oligonucleotide synthesis with common adaptor elements (Supplementary Table S1). These regulatory elements were demonstrated to have high levels of transcription in various cell lines (Schlabach et al., 2010) and tissues including neural tissues (Yaguchi et al., 2013; Carullo and Day, 2019). We paired each element with a unique 16 bp barcode. Since the elements have different sizes, we can detect proper AAV transduction or transcription using polymerase chain reaction (PCR) of genomic DNA or complementary DNA (cDNA), followed by visualization of 3 DNA bands with electrophoresis. Alternatively, these regulatory elements can drive transcription of a nuclear fluorophore which we visualize with immunofluorescence (Figure 1B).

#### Positive controls

We selected 10 positive controls from Nguyen et al. (2016) and 20 positive controls from Kheradpour et al. (2013) to maximize the RNA:DNA ratio, making the enhancer most likely to regulate expression within our assay. For the Nguyen *et al.* enhancers, the simulated neural cell samples were ignored in favor of those enhancers with a higher baseline expression rate. In all cases the positive control enhancers were identified in the cell type similar, but not identical to, the cell type or tissue of interest. We selected candidate brain enhancers from cultured mouse cortical neurons, candidate liver enhancers from HEPG2 cells, and candidate HMC3 enhancers from K562 cells.

#### Negative controls

We selected 10 negative control enhancer sequences that displayed very low RNA:DNA ratios in cultured mouse cortical neurons as previously published by Nguyen et al. (2016) In addition, we generated a set of 30 random sequence enhancers to create candidate negative control enhancers that were the same length as positive control enhancers including variations in GC content (10 each of 30%, 50%, and 70% GC).

#### Evaluating necessity of MEF2C binding for enhancer activity

MEF2C is a transcription factor that has been shown to play multiple roles in the cortex and striatum, including driving interneuron morphological maturation (Pai et al., 2020) and regulating cortical excitatory-inhibitory synapses (Harrington et al., 2016; Chen et al., 2016). Mef2c upregulation has been implicated in schizophrenia (Mitchell et al., 2018) and reduced vocalization abilities due to its role in repressing dendritic spine development in striatal neurons (Chen et al., 2016). These important roles of transcription factor MEF2C in the cortex and striatum suggest that the binding of this transcription factor may be necessary for enhancer activity. However, testing this hypothesis was previously infeasible due to the inability of cell lines to accurately represent the full dynamics of *in vivo* cortex and striatum transcriptional regulatory programs. Therefore, we designed sequences for our sysMPRA library to directly evaluate the necessity of the MEF2C motif for enhancer activity in the brain. In particular, we pinpointed potential brain-specific enhancers containing candidate MEF2C binding sites and designed sequences for comparing their activity with that of sequences lacking the identified MEF2C binding sites.

#### Cortical and striatal enhancers near genes associated with vocal learning

Vocal learning is a complex trait that has evolved independently in multiple clades of birds and mammals (Wirthlin et al., 2019), serving as a useful trait to study the genetic mechanisms involved in the evolution of fine-motor behavior and exploring the overall relationship between genotype and phenotype. Epigenomic data pertaining to candidate regulatory enhancers were used to design sequences for evaluating the effects of enhancer activity on vocal learning evolution. These candidate enhancers exhibit broad conservation across mammals and are situated in close proximity to genes associated with vocal learning and human speech disorders (Supplementary Methods). This approach facilitated the assessment of enhancer activity conservation in these regions among both vocal learners and non-learners.

#### Evaluating effects of AD-associated variants

The AD GWAS summary statistics were downloaded from Lambert et al. (2013) and the GWAS p-values were visualized alongside brain cell type-specific H3K27ac ChIP-seq signal tracks and peak calls derived from our previous work in the Integrated Genomics Viewer (IGV) (Ramamurthy et al., 2020; Robinson et al., 2011). H3K27ac is a histone mark associated with active enhancers and promoters (Creyghton et al., 2010). SNPs to be included in the array were selected based on multiple criteria. Fifteen SNPs were chosen that met one or more of the following criteria : (1) Having a significant multiple hypothesis corrected GWAS p-value, (2) Overlapping with H3K27ac peaks in neurons and microglia, with consideration of whether they are present in a H3K27ac signal dip (Ernst et al., 2011), (3) Being a sentinel SNP in the AD associated haplotype block or being in high linkage disequilibrium with a sentinel SNP, (4) Disrupting motifs for transcription factors crucial for neuronal and microglial function or highly expressed in neuron and microglia such as SPI1 (Pu.1), EGR1, MEF2, FOXA1, FOXA2 (Ward and Kellis), and (5) having an eQTL association with the expression of well-studied AD associated genes that are highly expressed in microglia, such as BIN1 (Nott et al., 2019) and SPI1 (Gjoneska et al., 2015b). In addition, 12 SNPs were selected that overlapped with human ortholog of a differential H3K27ac peak identified in the brain of the CK-p25 mouse model of AD (Gjoneska et al., 2015b). The identification of human orthologs for the mouse differential peaks was carried out using liftover with default settings (Kent et al., 2003). For each selected SNP, two MPRA enhancer sequences were incorporated into the array, with one sequence carrying the reference allele and the other carrying the alternative allele. Additionally, in certain instances where the SNP disrupted transcription factor binding (TFBS) motifs for AD-associated TFs (SPI1, EGR1, MEF2, FOXA1, and FOXA2), a third enhancer sequence was included, carrying a randomly shuffled version of the motif. The sequences for all included candidate enhancers were centered on the location of the SNP except for 10 candidates, where the sequences were centered elsewhere to ensure the full TFBS motif could be incorporated for sequence variants causing TFBS disruption.

### Experimental design

#### Plasmids

Three cross-tissue (CT) positive controls (Supplementary Table S1) were synthesized with strong viral regulatory elements for the MPRAct library and the g-blocks were ordered by Integrated DNA technologies (IDT). The sequence fragments for the insert library (MPRAi; Supplementary Table S2) were synthesized by Agilent Technologies. The MPRA Insert Library Template (Supplementary Figure S1) for both the MPRAi library and the CT positive controls followed a structured arrangement. This includes a common sequence for 5′ cloning, the candidate enhancer sequence, 27 bp of common sequence linker, 16 bp dedicated to the DNA barcode, and a common sequence for 3′ cloning. 5 pmol of MPRAi library was amplified with Herculase II Fusion Polymerase (Agilent Technologies, #600675) and purified with AMPure XP beads (Beckman Coulter, #A63881) at a ratio of 1.8x and subsequently eluted in Elution Buffer (EB, Qiagen #19086).

The amplified MPRAi insert library, the CT positive control gBlocks and the pAAV-Hsp68-nls/mCherry-MPRAe (pAAV-MPRAe) vector were digested with SgsI (Thermo Fisher Scientific, #FD 1894) and SfaAI (Thermo Fisher Scientific, #FD 2094) (Figure 1A; Supplementary Figure S1) and purified by using QIAEX II kit (Qiagen, # 28704). The linearized pAAV-MPRAe was dephosphorylated with Shrimp Alkaline Phosphatase (Affymetrix, #78390) at the 5′ ends to prevent religation of the vector. Then, the MPRAi library and each CT positive control insert was ligated with T4 DNA ligase (New England Biolabs, #M0202 S) into the digested pAAV-MPRAe vector and purified by using isopropanol DNA precipitation. The ligation reactions were transformed into MegaX DH10 B electrocompetent *E. coli* cells (Thermo Fisher Scientific, #C640003) and plated on LB medium supplemented with Ampicillin for selection. For each CT positive control, clones were selected, plasmids extracted (Qiagen, Cat: 27104) and purified and subsequently verified by sanger sequencing (Eurofins Genomics). The pAAV-Hsp68-nls/mCherry-MPRAct (pAAV-MPRAct) plasmid library was constructed by combining each CT positive control plasmid at equal concentrations. For the pAAV-Hsp68-nls/mCherry-MPRAi (pAAV-MPRAi) plasmid library, the transformants were split: (1) a small portion was used for assessing the transformation efficiency by plating on LB plates supplemented with Ampicillin and counting the colonies, (2) the rest of the transformants were inoculated in LB liquid medium supplemented with Ampicillin for selection and plasmid DNA was extracted utilizing the EndoFree Plasmid DNA prep kit (Qiagen, #12362, 12,381, or 12,391). For assessing the library complexity (enhancer and barcode coverage) the pAAV-MPRAi plasmid library was amplified with Nextera indexing primers and sequenced on the Miseq (Illumina) using the V2 300 paired end read cycle kit (2 × 151 base pair; eight base pair indexing reads, Illumina, MS-102-2002).

#### Cell lines

For MPRA library analysis, human embryonic microglial (HMC3) cells were purchased from the American Type Culture Collection (ATCC, #CRL-3304) and cultured according to the manufacturer’s guidelines. The AAV viral production was executed in AAVPro(R) human embryonic kidney 293 (293T) cells from Clontech (Takara Bio USA, #632273) and cultured according to manufacturer’s instructions.

HMC3 cells were seeded in a 100 mm dish and grown to approximately 80% confluent and transfected. Approximately 24 h after seeding, cells were transfected with 250 µg pAAV-MPRAi plasmid library DNA using transfection reagent FuGene 6 (Promega, #E5912) at a FuGene6:DNA ratio of 3:1. Cells were incubated at 37°C for 72 h, harvested from each dish, and subsequently centrifuged at 500 *g* for 5 min. Genomic DNA and total RNA were extracted by using DNeasy Blood and Tissue Kit (Qiagen, #69504) and RNeasy Mini Kit (Qiagen, cat #74104), respectively.

Adeno-associated virus (AAV) was produced in 293T cells according to previously published protocols (Jang et al., 2022; Huang et al., 2013) (Supplementary Methods). The virus was titered using the AAVpro Titration Kit (Takara Bio USA, #6233), aliquoted into LoBind tubes (Eppendorf, #0030108434), and finally stored at −80°C until further use.

#### Animals

All animal procedures were approved by Carnegie Mellon University Institutional Animal Care and Use Committee (IACUC). Molecular and imaging experiments were performed on 3–6 month old female and male C57BL/6J mice (The Jackson Laboratory, strain #000664). The pAAV-MPRAi library was injected into 8 mice (4 males and 4 females) for library sequencing, and 4 mice (1 male and 3 females) were used for immunofluorescence experiments (Figure 2). The pAAV-MPRAct library was injected into 2 female mice for immunofluorescence imaging experiments (Figure 2; Supplementary Table S3).

The mice were anesthetized using 1%–4% isoflurane until breathing slowed and the pedal reflex was no longer detected. A total of 7.73 × 10^11^ to 2.28 × 10^12^ vector genomes (vg) were injected into the retro-orbital cavity. Subsequent to the injections, the mice were administered 0.5% proparacaine hydrochloride ophthalmic solution for comfort and were closely monitored for any abnormalities or signs of distress post-procedure. The virus was incubated in the mice for 3–6 weeks and tissue was collected for downstream experiments.

For the library sequencing experiments, the animals were deeply anesthetized with isoflurane until a lack of pedal withdrawal was observed and euthanized by decapitation. Immediately following death, fresh tissues were harvested (Supplementary Figure S3). Brain tissue was sectioned with a Leica VT 1200 vibrating microtome at a thickness of 300 μm, staged in cold, oxygenated artificial cerebrospinal fluid (aCSF). The primary motor cortex (M1), prefrontal cortex, other frontal cortex (referred to as cortex throughout this paper), striatum, hippocampus, and hypothalamus from the brain sections were dissected and each brain region was divided into two tubes, flash frozen, and stored at −80°C until processing. Additionally, liver, testes, ovaries, lung, kidney, muscle, and heart were harvested immediately after decapitation and subsequently, tissues were minced into small pieces with a clean razor blade, divided into 2 tubes, flash frozen, and stored at −80°C until further use. From these tissues genomic DNA and total RNA were extracted using DNeasy Blood and Tissue Kit (Qiagen, #69504) and RNeasy Mini Kit (Qiagen, #74104), respectively. The DNA samples were stored at −20°C and the RNA at −80°C until further processing for DNA and RNA barcode sequencing.

For imaging experiments, mice were deeply anesthetized with isoflurane and confirmed with a negative toe-pinch response. Intraperitoneal urethane (50 mg/mL, Acros Organics, #A0378229) was administered, and cardio-thoracic perfusion was performed with 1x PBS followed by 4% paraformaldehyde (approximately 10 mL each). Subsequently, tissues were harvested and incubated in 4% paraformaldehyde for 4–12 h at 4°C. Following incubation, tissues were washed with 1x PBS to remove paraformaldehyde and stored in 1x PBS at 4°C until the tissues were ready to undergo processing for immunofluorescence staining and imaging.

#### Immunofluorescence staining and imaging

Tissues were sectioned on a Leica VT1000 S vibratome, sliced at 80 μm, and probed for nuclear mCherry expression using a standard immunohistochemistry protocol. Brain tissues were stained with primary anti-NeuN (Cell Signaling #94403, 1:500) or anti-mCherry (Cell Signaling, #43590, 1:500) and subsequently stained with secondary antibody AlexaFluor 488 (Thermo Fisher Scientific #A11029, 1:500) or AlexaFluor 594 (Cell Signaling, #8889, 1:500; Figure 2), respectively. Liver tissues were stained with 4′,6-diamidino-2-phenylindole (DAPI, Thermo Fisher Scientific #D1306) or primary anti-mCherry (Cell Signaling, #43590, 1:500) and followed with secondary antibody AlexaFluor 594 (Cell Signaling, #8889, 1:500). The tissue slices were mounted on glass slides (Fisher Scientific, Cat. #12-550-18) and coverslipped with ProLong Diamond Antifade Mountant (Thermo Fisher Scientific, #P36961). The tissues were imaged using a laser scanning confocal microscope (LSM 880, Carl Zeiss) with a Plan-Apochromat 10 × 1.3 NA objective and a spectral analysis camera. Laser lines 405 nm (DAPI), 488 nm (AlexaFluor 488) and 561 nm (AlexaFluor 594) were used with consistent settings across all samples. All images were processed and analyzed using Zeiss Zen Black software and ImageJ.

#### MPRA barcode library preparation from tissue and HMC3 cell lines

The barcode libraries were prepared for sequencing from the total RNA extracted from tissue. The RNA was treated with Turbo DNase I (Thermo Fisher Scientific, #AM2238) and SUPERase-In RNase Inhibitor (Thermo Fisher Scientific, #AM2694) according to the manufacturer’s instructions to remove any AAV vector genome DNA contamination. The RNA was purified with the RNeasy MinElute CleanUp Kit (Qiagen, #74104) and concentrations were quantified using a Qubit fluorometer (Thermo Fisher Scientific). Subsequently, a specific Reverse Transcription (RT) was performed on the RNA (up to 2.0 µg) by using the SuperScript IV enzyme (Thermo Fisher, #18090200) and a specific RT primer (GTA​CAA​GAA​AGC​TGA​ACG​AGA​AAC​G) complementary to the 3′ tail of the MPRA transcript, positioned before the SV40 polyadenylation signal. This was followed by an RNA denaturation treatment using 1 M NaOH, pH > 10 at 98°C for 20 min. The cDNA for each sample was purified by isopropanol precipitation with GlycoBlue Co-precipitant (Thermo Fisher Scientific, #AM9515) to help visualize the DNA pellet.

Both genomic DNA and cDNA were amplified with dual-indexing primers synthesized from Eurofins Genomics following the Illumina Nextera tagmentation format as previously published (Preissl et al., 2018) (Supplementary Table S5). For PCR amplification the NEBNextPhusion High-Fidelity PCR Master Mix (New England Biolabs) was used and all samples were purified and concentrated with the MinElute PCR Purification Kit (Qiagen, #28004). The quantity and quality of each sample was measured using the Qubit and Agilent TapeStation.

#### Sequencing

To obtain the initial library quality estimates and balance sample representation for deep sequencing, each sample was pooled and sequenced with 5% PhiX (Illumina, #FC-110-3,001) on the Illumina MiSeq system using a 150-cycle V3 Kit (Illumina, #MS-102-3,001). Prior to deeper sequencing, the library pool was rebalanced and thereafter, the libraries were sent for sequencing with a targeted number of reads per sample with 30% PhiX on two NovaSeq S4 flowcells (GenWiz by Azenta). Due to the extensive number of samples in the project, two separate NovaSeq experiments were conducted to achieve the desired sequencing depth across all samples. To mitigate possible batch effects, several high-quality and low-quality samples were included on each NovaSeq experiment, ensuring their repetition in each sample pool. Throughout all sequencing runs and intermediate steps, compliance to the Illumina and GenWiz guidelines was maintained.

### Computational analyses

#### Quantifying enhancer activity from MPRA libraries

To quantify enhancer activity, the sequence data underwent processing by removal of the low-quality samples (Supplementary Methods). Subsequently, the read counts showed high correlation across the two NOVA-Seq runs, with a median 0.99 RNA barcode correlation across technical replicates. The counts associated with barcodes were combined to generate matrices representing read counts per sample-enhancer combination. Next, MPRAnalyze (Ashuach et al., 2019) was employed with default settings to determine the raw transcriptional activity (alpha) and the normalized transcriptional activity (MAD Score) of each enhancer. MPRAnalyze focuses on results that are consistent across animals. That statistical model can either aggregate across animals or across tissues. We chose to aggregate across animals. This analysis was conducted at multiple levels: (1) per sample, (2) per tissue, and (3) per tissue type (brain, liver, HMC3). The results reported are only those that are consistent across animal replicates. Additionally, the significance enhancer activity was calculated relative to the negative control levels (Figure 3A).

#### Comparison to machine learning model predictions

To predict enhancer activity for sysMPRA brain and liver candidate enhancers, we utilized our prediction models trained by open chromatin data of the brain and liver from multiple species (models 8–9), as previously published by our group (Kaplow et al., 2022). Since our models require 500 base pair sequences, while sysMPRA enhancers were only 120 base pairs, we employed the bioinformatics program Biopython version 1.74 (Cock et al., 2009) to extend each sequence by 190 base pairs of Ns on both sides. This resulted in the 500 base pair sequence with the sysMPRA candidate enhancer positioned in the center. These sequences were analyzed by using our models, which were trained using Keras version 1.2.2 (Chollet, 2015), to generate predictions for each sysMPRA candidate enhancer sequence as well as its reverse complement. The predictions obtained from the forward and reverse complement candidate enhancer sequences were averaged and subsequently compared with the enhancer activity measured through our sysMPRA technology.

#### Disruptions of transcription factor binding sites and SNPs

To analyze disruptions of transcription binding factor sites and SNPs, the MAD score was calculated using MPRAnalyze. For the MEF2C transcription factor sequences designed at MEF2C transcription factor binding sites, sequences were compared with altered versions, where either the MEF2 transcription factor binding site or the site along with the surrounding region was shuffled. Cases where the disruption of the MEF2 transcription factor binding site motif affected enhancer activity were identified through a paired t-test conducted across all samples in the primary motor cortex, other cortex, and striatum (Supplementary Table S7). Similarly, the enhancer activities of various alleles and disruptions caused by candidate Alzheimer’s disease (AD)-associated mutations were assessed through a paired t-test of the MAD scores (Supplementary Table S8). This analysis was carried out using HMC3 cells and neural tissues implicated in AD predisposition and progression, with the exclusion of liver tissue from the comparison.

## Data Availability

All raw and processed sequencing data generated in this study have been submitted to the NCBI Gene Expression Omnibus https://www.ncbi.nlm.nih.gov/geo/query/acc.cgi?acc=GSE223307 under accession number GSE223307 (token mpetgwwovxahryd). Code for computational analyses, including arrayProc.2.1.1.py, can be found at Github: https://github.com/pfenninglab/sysMPRA and Zenodo: DOI: 10.5281/zenodo.7527429.
