# Supplementary figures and images for: An in vivo systemic massively parallel platform for deciphering animal tissue-specific regulatory function
Source: Front Genet. 2025 Apr 9;16:1533900. doi: 10.3389/fgene.2025.1533900 (PMC12016043; doi:10.3389/fgene.2025.1533900)

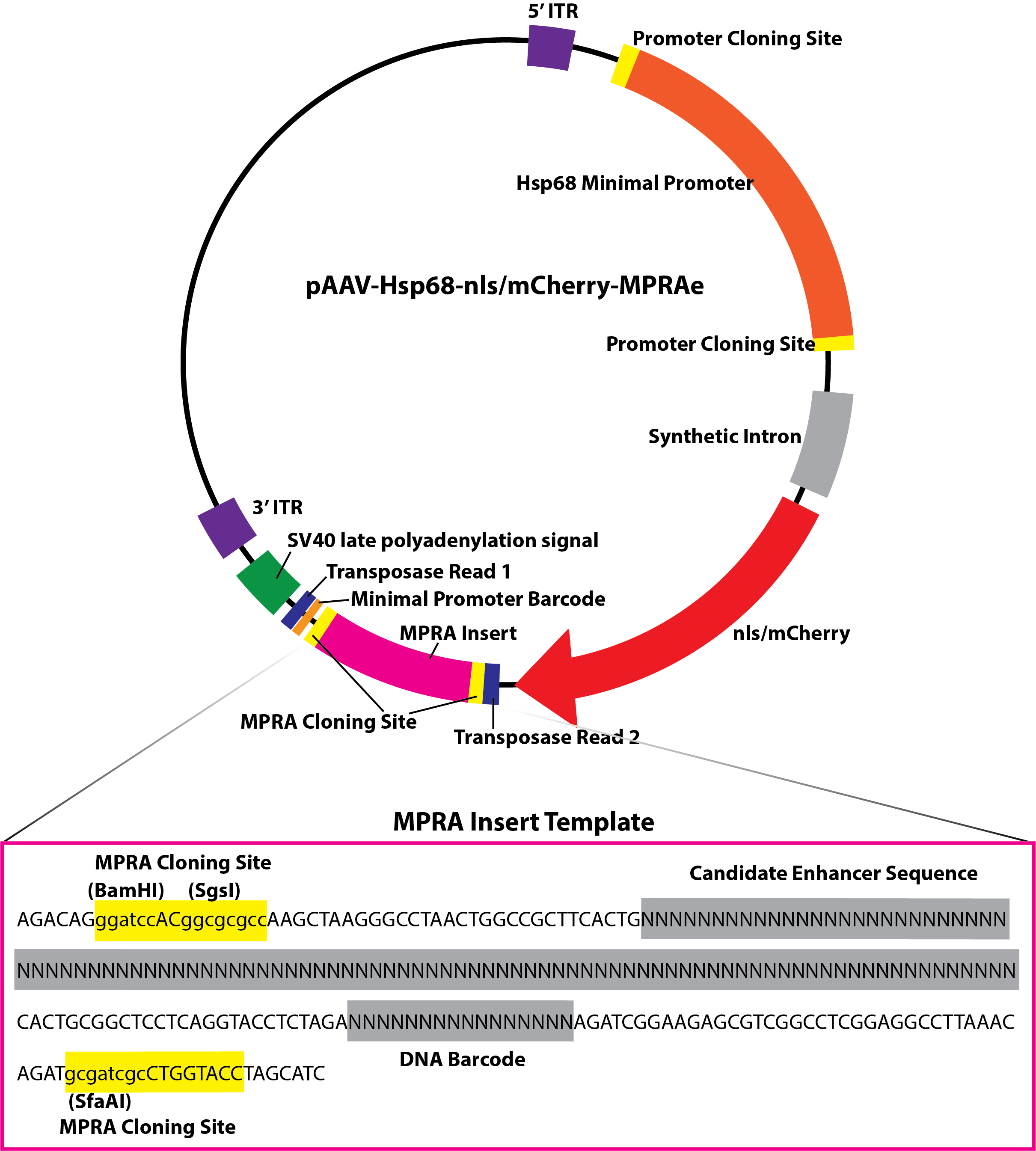

Supplement: Supplementary file 2 [file Image1.tiff]

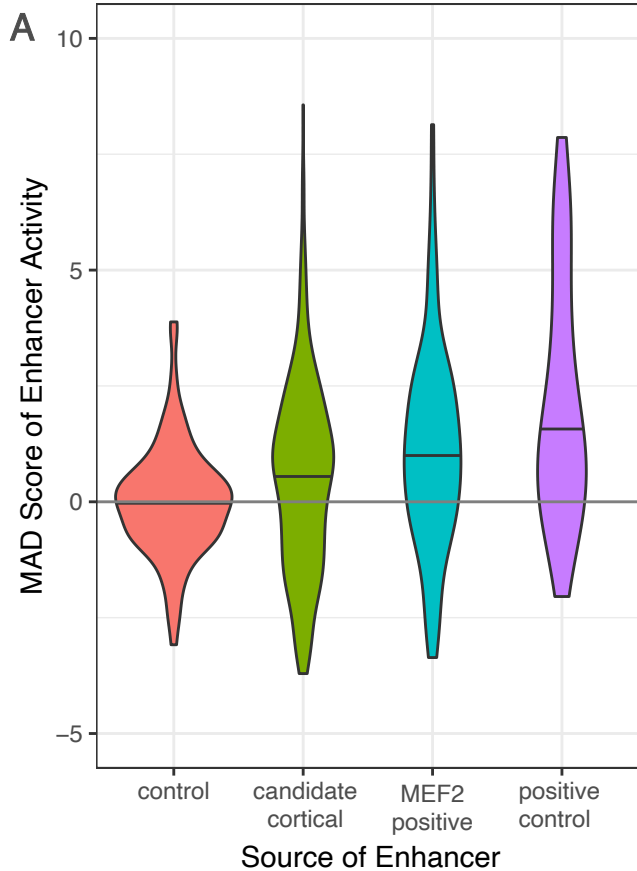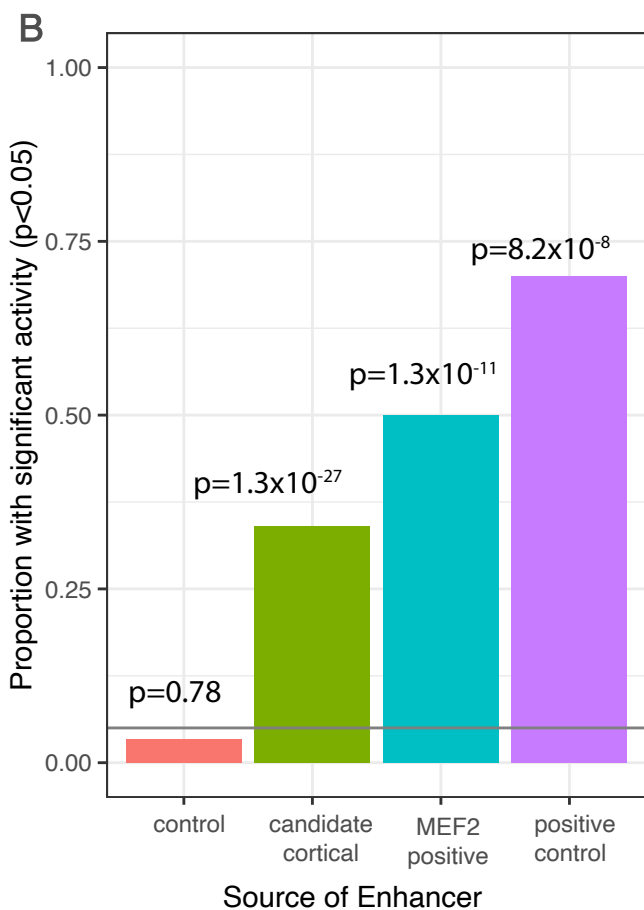

Supplement: Supplementary file 3 [file Image5.pdf]

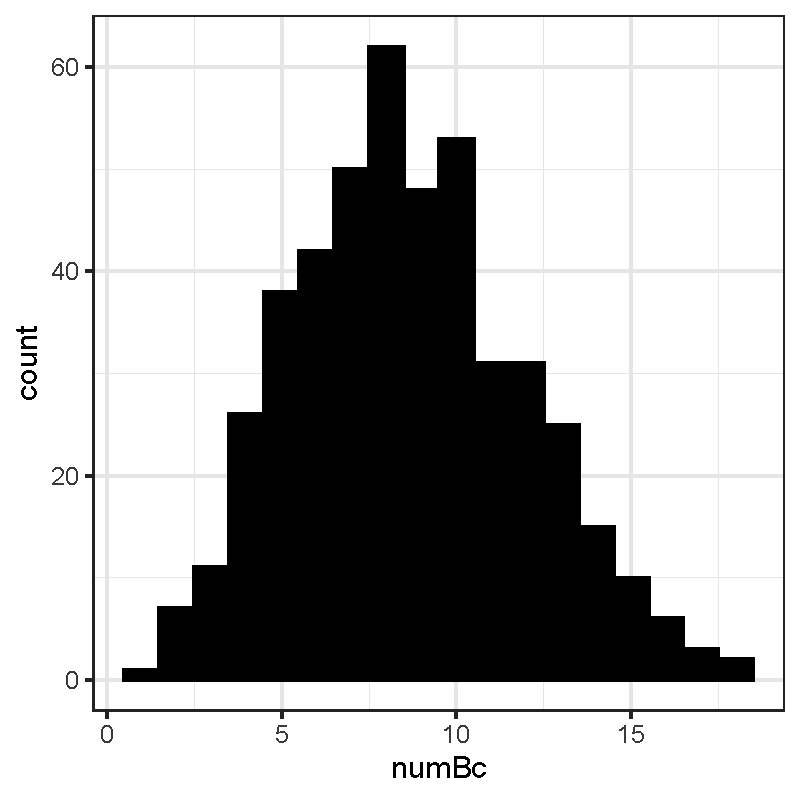

Supplement: Supplementary file 6 [file Image2.tif]

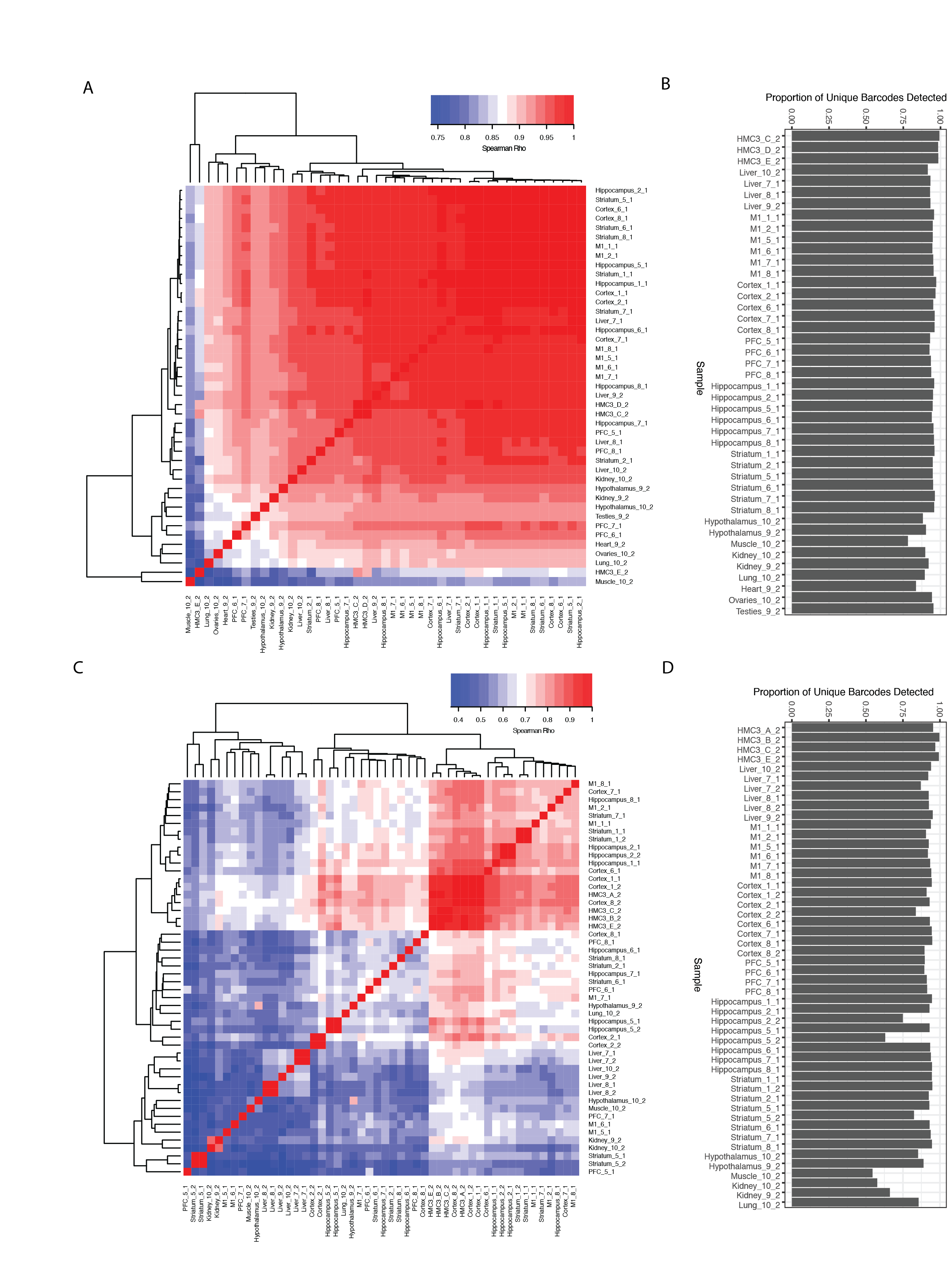

Supplement: Supplementary file 8 [file Image4.png]

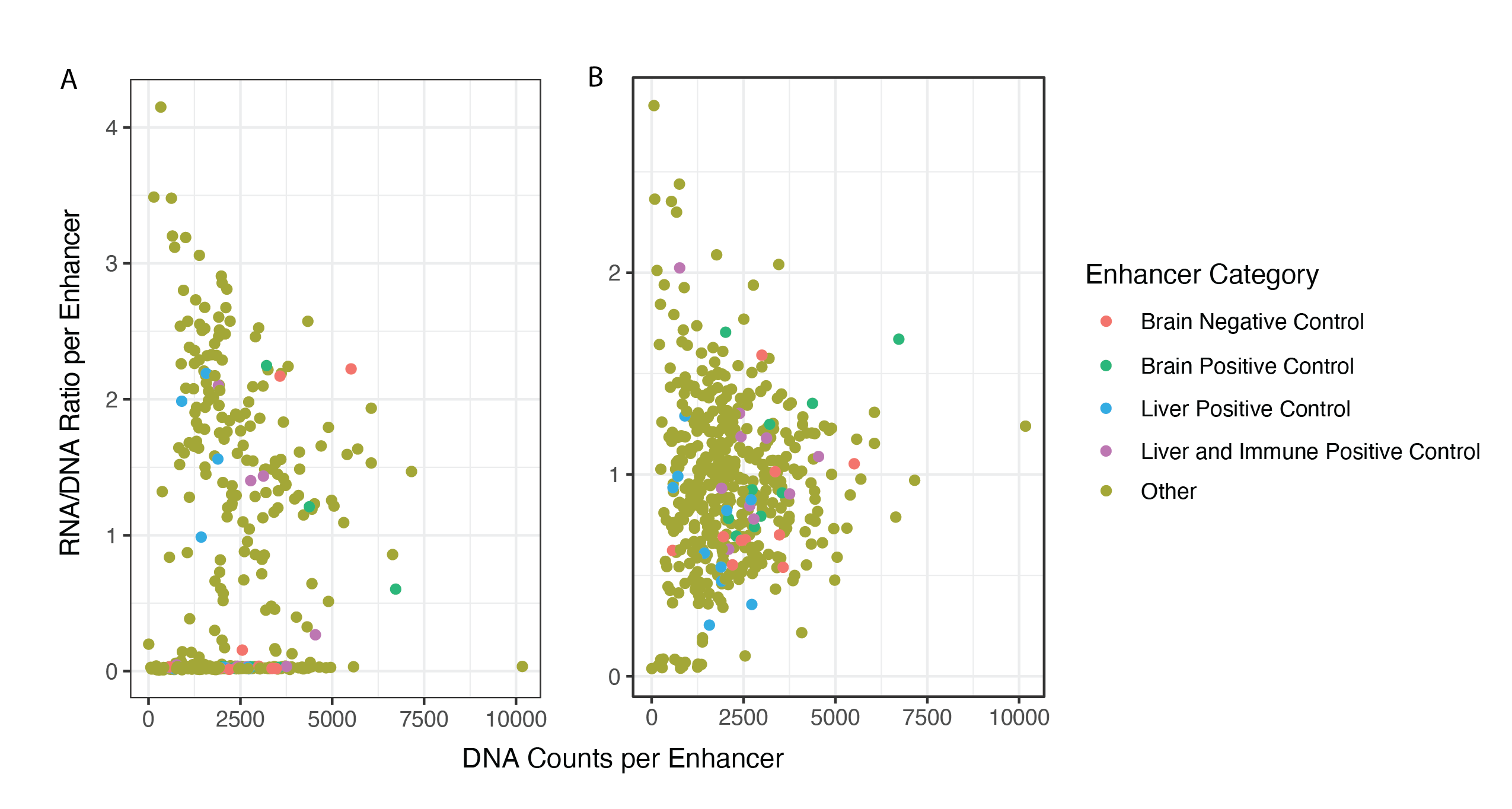

Supplement: Supplementary file 11 [file Image6.png]

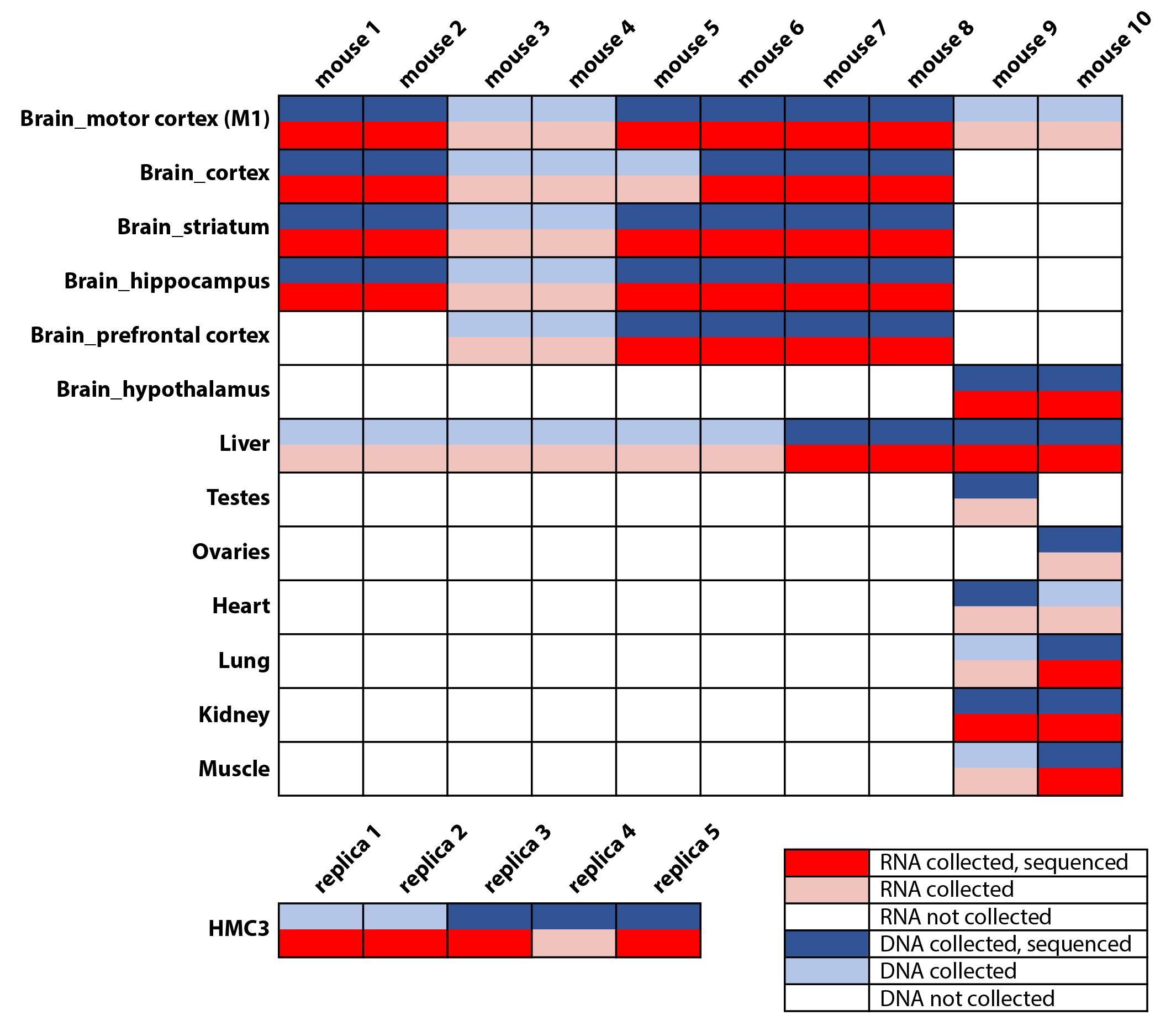

Supplement: Supplementary file 13 [file Image3.png]
