# Supplementary material for: An in vivo systemic massively parallel platform for deciphering animal tissue-specific regulatory function
Source: Front Genet. 2025 Apr 9;16:1533900. doi: 10.3389/fgene.2025.1533900 (PMC12016043; doi:10.3389/fgene.2025.1533900)
Supplement: Supplementary file 15 [file DataSheet1.docx]

**An *in vivo* systemic massively parallel platform for deciphering animal tissue-specific regulatory function**

**Running title: systemic massively parallel reporter assay**

**Ashley R. Brown^1,2^, Grant A. Fox^1,2,4,#^, Irene M. Kaplow^1,2,3,#^, Alyssa J. Lawler^2,3,5^, BaDoi N. Phan^1,2,6^, Lahari Gadey^1,2^, Morgan E. Wirthlin^1,2,7^, Easwaran Ramamurthy^1,2^, Gemma E. May^3^, Ziheng Chen^3^, Qiao Su^1,2^, C. Joel McManus^3^, Robert van de Weerd^1,2^, and Andreas R. Pfenning^1,2,3, *^**

Departments of ^1^Ray and Stephanie Lane Department of Computational Biology, ^2^Neuroscience Institute, and ^3^Department of Biological Sciences, Carnegie Mellon University, Pittsburgh, PA 15213, USA; ^4^Current affiliation: College of Medicine, University of Kentucky, Lexington, KY 40506, USA; ^5^Current affiliation: Stanley Center for Psychiatric Research, Broad Institute, Cambridge, MA 02142, USA; ^6^Medical Scientist Training Program, University of Pittsburgh School of Medicine, Pittsburgh, PA 15213, USA; ^7^Current affiliation: Allen Institute for Brain Science, Seattle, WA 98109, USA;

^#^ Authors contributed equally to this work.

* To whom correspondence should be addressed. Email: apfenning@cmu.edu

**SUPPLEMENTARY FIGURES**

**Supplementary Figure 1 - MPRA plasmid design**

Cartoon representation of the candidate enhancer library MPRA plasmid. The top panel depicts the map of pAAV-Hsp68-nls/mCherry-MPRAe (pAAV-MPRAe) plasmid, providing an overview of the design features. The plasmid is based on the PHP.eB and AAV 2 serotypes, with its elements listed in a 5' to 3' orientation. It commences with the 5' inverted terminal repeat sequences (5' ITR, purple), followed by the Hsp68 minimal promoter (orange) with flanking promoter cloning sites (yellow), a synthetic intron, the fluorescent reporter mCherry with the nucleus localization signal (NLS), Illumina Transposase Read 2 sequence, the MPRA insert library (candidate enhancers and DNA barcodes, pink) with flanking cloning sites (yellow), a minimal promoter DNA barcode unique for the core vector design elements and library type (orange), Illumina Transposase Read 1 sequence, the SV40 late polyadenylation signal (polyA tail, green), and the 3' ITR. The bottom panel shows a detailed sequence view of the MPRA insert template (pink) utilized in the library design. The listed sequences remain constant, while the gray shaded regions represent the variable elements, encompassing the candidate enhancer and DNA barcode sequences. The yellow highlighted sections denote the cloning sites, featuring restriction enzyme digestion sequences in lowercase letters within the MPRA cloning sites.

**Supplementary Figure 2 - Histogram of numbers of barcodes detected for each candidate enhancer in the final library after the cloning procedure.**

**Supplementary Figure 3 - Sample collection and sequencing matrix**. Ten mice were injected (retro-orbital) with the MPRAi library and various tissues were extracted. We transfected the plasmid library into HMC3 cells for 5 separate replicates. This table represents sample quality, sequencing status and collected tissue types: Dark red, RNA was collected from sample and library was sequenced; Light red, RNA was collected, but not sequenced due to low quality; Dark blue, DNA was collected from sample and sequenced; Light blue, DNA was collected, but not sequenced due to low quality; White, RNA and/or DNA was not collected.

**Supplementary Figure 4** **- Correlation of DNA and RNA barcode measurements across tissues *in vivo*.** The Spearman correlation is assessed across all samples for the plasmid DNA barcode reads (A) and for the RNA barcode expression reads (C). For each sample, the proportion of unique barcodes detected in that sample is plotted relative to the barcodes detected across all samples (3,983) for both the DNA (B) and for RNA (D).

**Supplementary Figure 5 - Comparing candidate and control brain enhancers.** We calculated the MAD score (A) and the MAD p-value (B) for enhancers positive and negative control enhancers as well as the candidate enhancers chosen based on brain epigenomics and MEF2C. The candidate enhancers were substantially more likely than expected to drive significant activity in the brain (B).

**Supplementary Figure 6** **- Enhancer dropout observed in low quality samples that are excluded from the analysis**. As examples two samples are displayed, (A) the striatum from animal 2 and (B) animal 5. The RNA:DNA ratio per enhancer is plotted relative to the number of DNA barcode reads (counts) per enhancer detected from the enhancer plasmid DNA (thousands) in the assay. Each enhancer is colored based on its design group (enhancer category). In the striatum sample from animal 2, there are multiple positive controls that have no RNA reads despite having large numbers of DNA reads, and, even though most of the other enhancers were designed to be active in the striatum and have many DNA reads, many have RNA/DNA ratios close to 0. In contrast, in the striatum from animal 5, all of the positive controls have RNA/DNA ratios that are greater than 0, and many of the other enhancers with large numbers of DNA reads also have large RNA/DNA ratios. Legend abbreviations are defined in the caption of **Table S2**.

**SUPPLEMENTARY TABLES**

**Supplementary Table 1** - **Test MPRAct Library Sequences.**

The sequences for the cross-tissue positive control library (MPRAct). The columns list the synthesized DNA sequence for the cloning procedure and the enhancer sequence as well as the 16 bp unique barcode sequence.

**Supplementary Table 2** - **MPRAi Library Sequence and Annotation.**The columns list the enhancer sequences, barcodes, synthesized DNA sequences, source of the sequences, and whether or not the sequences were detected at RNA and DNA levels. The sequence sources are categorized based on their relevance to the intended questions: **AD** (sequence with Alzheimer’s disease variant); **GC** (randomly generated sequences with specific GC contents); **MEF2C** (MEF2C-bound, MEF2C-bound with motif shuffled, or ortholog of MEF2C-bound sequence); **VL** (sequence implicated in vocal learning evolution); **PK_liv**

(sequences with liver specific enhancer activity from [^1^](https://paperpile.com/c/Nrmxau/2EZ6t)); **PK_livImm** (sequences with enhancer activity in liver and K562 from [^1^](https://paperpile.com/c/Nrmxau/2EZ6t); **TN_cort** (sequences with high baseline activity in cultured cortical neurons from [^2^](https://paperpile.com/c/Nrmxau/oTeqt)); **TN_neg** (sequences with low baseline activity in cultured cortical neurons from [^2^](https://paperpile.com/c/Nrmxau/oTeqt)).

**Supplementary Table 3** - **Sample and Quality Control Information.**

For each sample, the number of reads with the barcode at both DNA and RNA levels was calculated. The proportion of reads containing a recognizable barcode and restriction enzyme site was also determined.

**Supplementary Table 4** - **DNA Barcode Counts.**

The number of each barcode, measured at the DNA level.

**Supplementary Table 5** - **RNA Barcode Counts.**

The number of each barcode, measured at the RNA level.

**Supplementary Table 6** - **Machine Learning Model Predictions.**

For each enhancer, the predicted activity or open chromatin level across the different cell types and tissue assayed is provided.

**Supplementary Table 7** - **MEF2 Binding Site Disruption.**

Results of paired t-tests between each candidate MEF2-binding enhancer and the versions where the MEF2 motif is disrupted through nucleotide shuffling.

**Supplementary Table 8** - **Disruption of AD-Associated SNPs at Candidate Enhancers.**

Results of paired t-tests between each of the reference and alternate alleles of candidate enhancers associated with AD predisposition.

**SUPPLEMENTARY METHODS**

**Array design**

Designing sequences for evaluating necessity of MEF2C binding for enhancer activity

To identify candidate brain-specific enhancers, the ATAC-Seq / DNase-Seq Pipeline [^3^](https://paperpile.com/c/Nrmxau/Kle2e) with mm10 [^4^](https://paperpile.com/c/Nrmxau/klRcI) and default parameters except for an irreproducible discovery rate (IDR) threshold of 0.1 [^5^](https://paperpile.com/c/Nrmxau/MTwwq) was used to call peaks in each mouse motor cortex and striatum replicate from GSE161374 [^6^](https://paperpile.com/c/Nrmxau/7JRop). Liver was used as our reference outgroup tissue for defining brain-specific potential candidate enhancers. The data processing procedure was repeated for the postnatal liver ATAC-seq data from GSE172770 [^7–9^](https://paperpile.com/c/Nrmxau/GCJNe+pOHyN+zMDjF). For each of the cortex and striatum, we concatenated the peaks from each replicate with the peaks from the liver replicates. Next, all peaks within fifty base pairs of each other were merged using mergeBed from bedtools version 2.26.0 with option -d 50 [^10^](https://paperpile.com/c/Nrmxau/sJlSr). Then, parts of peaks overlapping protein-coding transcripts from mouse GENCODE version 15 [^11^](https://paperpile.com/c/Nrmxau/GD5Lm) were removed using subtractBed from bedtools version 2.26.0 with default parameters [^10^](https://paperpile.com/c/Nrmxau/sJlSr), 200 bp were added to the end of each peak using slopBed from bedtools version 2.26.0 with option -b 200 [^10^](https://paperpile.com/c/Nrmxau/sJlSr), and the numbers of reads in each of these regions were obtained. Next, for both the motor cortex and striatum, peaks that were significantly stronger in each brain region relative to the liver were obtained using DESeq2 with default parameters [^12^](https://paperpile.com/c/Nrmxau/wY1pz). Our criteria for the cutoffs were fold-change > 1 and adjusted p-value < 0.05. The peaks that are significantly stronger in the motor cortex relative to the liver that overlapped peaks significantly stronger in the striatum relative to the liver were obtained using intersectBed from bedtools version 2.26.0 with setting -wa [^10^](https://paperpile.com/c/Nrmxau/sJlSr). After that, the sequences of the subset of likely enhancer peaks were obtained by using closestBed from bedtools version 2.26.0 with options -d -t first, along with the protein-coding transcripts from mouse GENCODE version 15 [^11^](https://paperpile.com/c/Nrmxau/GD5Lm). Peaks less than 20kb away from the closest transcript were removed. The sequences of the remaining peaks were then obtained using fastaFromBed from bedtools version 2.26.0 [^10^](https://paperpile.com/c/Nrmxau/sJlSr). The dinucleotide content of these sequences was obtained using fasta-get-markov from the MEME Suite with -m 1 [^13^](https://paperpile.com/c/Nrmxau/5Nvch).

To identify candidate MEF2C binding sites in the brain, MEF2C cortical neuron ChIP-seq data from GSM1629386 and the corresponding input data from GSM162938 [^14^](https://paperpile.com/c/Nrmxau/8YXZG) were downloaded. This data was processed using the AQUAS Transcription Factor and Histone ChIP-Seq processing pipeline [^15^](https://paperpile.com/c/Nrmxau/NRzHL) with mapping reads to mm10 [^4^](https://paperpile.com/c/Nrmxau/klRcI) and default parameters. The data was high-quality (NSC = 1.09, RSC = 1.20) [^7,16^](https://paperpile.com/c/Nrmxau/eA1wl+GCJNe), yielding 19,418 IDR [^5^](https://paperpile.com/c/Nrmxau/MTwwq) reproducible peaks across pseudo-replicates, where the pseudo-replicates were created by dividing the mapped reads into two groups because only one biological replicate was available. Next, MEF2C motifs within our brain-specific candidate enhancers were identified by running FIMO [^17^](https://paperpile.com/c/Nrmxau/Q8cbH) on their sequences with the background set to their dinucleotide content and the motif set to the MEF2C motif with ID M4467_1.02 from CIS-BP [^18,19^](https://paperpile.com/c/Nrmxau/84RII+F3TzZ).Then, the MEF2C peaks that intersected with the motif hits were identified using intersectBed from bedtools version 2.26.0 with options -wa and -wb [^10^](https://paperpile.com/c/Nrmxau/sJlSr); these peaks served as our candidate MEF2C binding sites within our brain-specific candidate enhancers.

Candidate MEF2C binding sites overlapping brain-specific candidate enhancers were prioritized if their brain-specific enhancer activity was especially likely or unlikely to be conserved in other mammals. To evaluate this, H3K27ac ChIP-seq regions from human and macaque cortex (occipital pole, precentral gyrus, and prefrontal cortex), striatum (caudate nucleus and putamen) [^20^](https://paperpile.com/c/Nrmxau/3YMkS), and liver [^21^](https://paperpile.com/c/Nrmxau/k4eKO) were obtained by processing the data using the AQUAS Transcription Factor and Histone ChIP-Seq processing pipeline with option -type histone [^15^](https://paperpile.com/c/Nrmxau/NRzHL). All datasets were high-quality, with NSC > 1.05 and RSC > 0.8 for every replicate [^7,16^](https://paperpile.com/c/Nrmxau/eA1wl+GCJNe). Next, for each of the human and macaque datasets, the peaks from different tissues and different biological replicates were merged and extended using the same methodology employed for open chromatin. Then, the number of reads in each species, tissue, and biological replicate combination in each histone modification region were identified using featureCounts with default parameters [^22^](https://paperpile.com/c/Nrmxau/8Vggq). Subsequently, in each of the human and macaque datasets, these read counts were used to identify regions exhibiting significantly higher H3K27ac ChIP-seq signal in the brain relative to the liver by employing DESeq2 with default parameters and alpha = 0.05 [^12^](https://paperpile.com/c/Nrmxau/wY1pz). Then, the macaque H3K27ac ChIP-seq regions were mapped to the human genome using liftOver [^23^](https://paperpile.com/c/Nrmxau/n1FvP). We obtained the following subsets of the human orthologs: 1) regions whose macaque orthologs have significantly stronger H3K27ac ChIP-seq in macaque brain compared to macaque liver that overlap human H3K27ac ChIP-seq regions are also significantly stronger in brain than in liver and 2) regions whose macaque orthologs do not have any stronger in macaque brain H3K27ac ChIP-seq than macaque liver H3K27ac ChIP-seq that overlap human brain H3K27ac ChIP-seq regions for which the H3K27ac is not any stronger in brain than in liver. These subsets were identified by using intersectBed from bedtools version 2.26.0 [^10^](https://paperpile.com/c/Nrmxau/sJlSr) with options -wa (-a was the human regions and -b was the human orthologs of macaque regions) and -u.

To leverage the differential H3K27ac ChIP-seq data to prioritize candidate MEF2C binding sites, the candidate MEF2C binding site peak summits overlapping our brain-specific candidate enhancers were also obtained and mapped to the human hg38 [^24^](https://paperpile.com/c/Nrmxau/Wd20b) assembly using liftOver [^25^](https://paperpile.com/c/Nrmxau/YoR7I). Then, the subset of these summit human orthologs overlapping human H3K27ac ChIP-seq regions that are significantly stronger in brain than in liver and human orthologs of macaque H3K27ac ChIP-seq regions that are significantly stronger in brain than in liver were obtained using intersectBed from bedtools version 2.26.0 [^10^](https://paperpile.com/c/Nrmxau/sJlSr) with options -wa and -u. Additionally, the subset of these summit human orthologs overlapping H3K27ac ChIP-seq regions that are not any stronger in brain than in liver and human orthologs of macaque H3K27ac ChIP-seq regions that are not any stronger in macaque brain than in liver were obtained in the same way.

Next, candidate MEF2C binding sites that are especially highly conserved were included, as conservation has been associated with function [^26–30^](https://paperpile.com/c/Nrmxau/7GT85+hTGK2+DrrCT+eoVZi+vJj5d). To include highly conserved MEF2C sites, the MEF2C peak summits at the candidate MEF2C binding sites in mouse brain-specific open chromatin were mapped to the human hg19 assembly [^31^](https://paperpile.com/c/Nrmxau/ddkv0) using liftOver [^25^](https://paperpile.com/c/Nrmxau/YoR7I). Then, these MEF2C peak summit orthologs were mapped to the zebra finch taeGut2 assembly [^32^](https://paperpile.com/c/Nrmxau/fulzH) using liftOver [^25^](https://paperpile.com/c/Nrmxau/YoR7I), thereby identifying peak summits that can be mapped between mammals and birds. Next, the zebra finch orthologs were mapped back to hg19 using liftOver [^25^](https://paperpile.com/c/Nrmxau/YoR7I), and the hg19 orthologs were mapped to hg38 [^31^](https://paperpile.com/c/Nrmxau/ddkv0) using liftOver [^25^](https://paperpile.com/c/Nrmxau/YoR7I). The different groups of human orthologs were combined and mapped back to the mouse mm10 assembly [^4^](https://paperpile.com/c/Nrmxau/klRcI) using liftOver [^25^](https://paperpile.com/c/Nrmxau/YoR7I).

These candidate MEF2C binding sites were further narrowed down to select those that are non-exonic, have a confident MEF2C motif match within sixty base pairs of their summits, and are near a gene known to be involved in the brain. Specifically, subtractBed from bedtools version 2.26.0 [^10^](https://paperpile.com/c/Nrmxau/sJlSr) was used to remove any of these candidate MEF2C binding sites that overlapped a mouse protein-coding exon from the mouse Gencode version 15 annotation [^11^](https://paperpile.com/c/Nrmxau/GD5Lm). Next, the sequences of the remaining candidate MEF2C binding site peak summits plus or minus sixty base pairs were obtained, where a total length of 120 base pairs was chosen because the candidate enhancers for the MPRA were 120 base pair sequences. This was achieved using fastaFromBed from bedtools version 2.26.0 [^10^](https://paperpile.com/c/Nrmxau/sJlSr). Then, FIMO [^17^](https://paperpile.com/c/Nrmxau/Q8cbH) was run on these sequences with the same settings as before. The candidate MEF2C binding sites that had a MEF2C motif hit within sixty base pairs of the summit with p-value < 0.0001 were selected. This resulted in the identification of sixty-nine candidate MEF2C binding sites.

For each of the sixty-nine candidate MEF2C binding sites, two shuffled versions of each sequence were created, where, for the first, the region matching the MEF2C motif hit was shuffled and, for the second, the region matching the MEF2C motif hit plus or minus five base pairs was shuffled. First, sequences with the shuffled MEF2C motif hits within sixty base pairs of the summits of the remaining candidate MEF2C binding sites were shuffled by obtaining the motif hit sequences with fastaFromBed from bedtools version 2.26.0 [^10^](https://paperpile.com/c/Nrmxau/sJlSr), shuffling them by running fasta-shuffle-letters from the MEME suite with options -copies 500 and -dna [^13^](https://paperpile.com/c/Nrmxau/5Nvch), and creating a sequence for each remaining candidate MEF2C binding site, shuffle combination in which the MEF2C motif hit was replaced with one of the shuffles. Next, motif hits within these modified sequences were identified by running FIMO [^17^](https://paperpile.com/c/Nrmxau/Q8cbH) on them with the same settings as before with one exception: All motifs from CIS-BP version 1.02 from human, macaque, mouse, and zebra finch were included. The results from FIMO were used to identify motif hits within the shuffled sequences that overlapped motif hits in the original sequences by running intersectBed with options -wa and -u from bedtools version 2.26.0 [^10^](https://paperpile.com/c/Nrmxau/sJlSr). Then, for each of the sixty-nine candidate MEF2C binding sites, the shuffled sequence with the highest motif hit p-value (worst MEF2C motif match) in the region corresponding to the MEF2C motif hit in the original sequence was selected. This process was repeated for MEF2C motif hits plus or minus five base pairs.

From the initial set of sixty-nine candidate MEF2C binding sites, sequences meeting certain criteria were filtered out. These included: (1) those with multiple motif hits with p-value < 0.0001, (2) those whose closest gene protein-coding gene was over 350 kb away, (3) those whose most significant motif hit p-value in the selected shuffled MEF2C motif hit was lower than the most significant MEF2C motif hit p-value in the original sequence, and (4) a subset of the candidate MEF2C binding sites that were initially selected only because their summits mapped from mouse to zebra finch. Specifically, the subset of candidate MEF2C binding sites initially selected solely due to their summits mapped to zebra finch that were over 100kb from the nearest gene were removed. Distances between candidate MEF2C binding sites and genes were determined using closestBed from bedtools version 2.26.0 with option -d [^10^](https://paperpile.com/c/Nrmxau/sJlSr) and the protein-coding transcripts from mouse GENCODE version 15 [^11^](https://paperpile.com/c/Nrmxau/GD5Lm). Then, those that overlapped candidate enhancers that decrease in activity in response to DNA damage, where those candidate enhancers were mapped from mm9 to mm10 using liftOver [^25^](https://paperpile.com/c/Nrmxau/YoR7I) and our MEF2C binding sites that overlapped them were identified using intersectBed from bedtools version 2.26.0 with options -wa and -u [^10^](https://paperpile.com/c/Nrmxau/sJlSr), were kept. Additionally, an additional random fourteen out of the eighteen remaining candidate MEF2C binding sites were kept. Finally, all selected candidate MEF2C binding sites that overlapped IDR reproducible motor cortex peaks from seven-week-old mice that were over 1 kb long were removed, as we thought these would be unlikely to have enhancer activity without their surrounding sequence. These overlapping peaks were identified using intersectBed from bedtools with options -wa and -wb [^10^](https://paperpile.com/c/Nrmxau/sJlSr).

The orthologs of our final set of twenty eight candidate MEF2C binding sites in human, macaque, zebra finch, and Egyptian fruit bat were obtained. The human and macaque orthologs were obtained by mapping the final set of candidate MEF2C binding sites to the human hg38 [^31^](https://paperpile.com/c/Nrmxau/ddkv0) and macaque rheMac8 [^33^](https://paperpile.com/c/Nrmxau/o0YsX) assemblies, respectively, using liftOver [^25^](https://paperpile.com/c/Nrmxau/YoR7I). The zebra finch orthologs were obtained by mapping the human orthologs to the human hg19 assembly [^24^](https://paperpile.com/c/Nrmxau/Wd20b) with liftOver [^25^](https://paperpile.com/c/Nrmxau/YoR7I) and then mapping the outputs from that to the zebra finch taeGut2 assembly [^32^](https://paperpile.com/c/Nrmxau/fulzH) with liftOver. The sequences of the mouse, human, macaque, and zebra finch orthologs were obtained using fastaFromBed from bedtools version 2.26.0 [^10^](https://paperpile.com/c/Nrmxau/sJlSr). The Egyptian fruit bat orthologs were obtained by finding the Egyptian fruit bat sequences in the Raegyp2.0 assembly [^34^](https://paperpile.com/c/Nrmxau/9p0yA) that corresponded to the mouse sequences with BLAT [^35^](https://paperpile.com/c/Nrmxau/XPZ8e), as there were no liftOver chains mapping mammalian or avian assemblies to an Egyptian fruit bat assembly. The sequences of all of these orthologs as well as the corresponding shuffled sequences were included in the MPRAi library.

Designing sequences to evaluate the enhancer activity conservation of cortical and striatal enhancers near genes associated with vocal learning

First, a set of 57,179 candidate cortical enhancers were generated through intersecting (1) frontal cortex and middle frontal gyrus DNase peaks (ENCODE IDs ENCSR000EIY, ENCSR000EIK, and ENCSR318PRQ) [^8^](https://paperpile.com/c/Nrmxau/pOHyN), which were from re-processing the data using the ATAC-Seq / DNase-Seq Pipeline [^3^](https://paperpile.com/c/Nrmxau/Kle2e) with default settings except for -enable_idr -dnase_seq; (2) motor cortex H3K27ac ChIP-seq regions [^20^](https://paperpile.com/c/Nrmxau/3YMkS), which were from re-processing the data as described above; and (3) a series of brain and liver candidate regulatory enhancers found to be conserved across a wide array of mammalian species [^20,21,36^](https://paperpile.com/c/Nrmxau/k4eKO+3YMkS+CLNpf). Second, a set of 53,401 candidate striatal enhancers were generated through intersecting (1) DNase peaks derived from human striatum (ENCODE IDs ENCSR015BGH and ENCSR493VDS) [^8^](https://paperpile.com/c/Nrmxau/pOHyN), which were from re-processing the data as described in our previous work [^37^](https://paperpile.com/c/Nrmxau/f57O6); (2) H3K27ac ChIP-seq regions derived from human caudate and putamen [^20^](https://paperpile.com/c/Nrmxau/3YMkS), which were from re-processing the data as described above; and (3) a series of brain and liver candidate regulatory enhancers found to be conserved across a wide array of mammalian species [^20,21,36^](https://paperpile.com/c/Nrmxau/k4eKO+3YMkS+CLNpf). 39,307 of the 71,273 total candidate regulatory enhancers from both sets (55%) overlapped H3K27ac ChIP-seq regions and DNase peaks in both cortex and striatum and were conserved across multiple mammalian species.

Out of these candidate enhancers, a subset of enhancers proximal to genes previously shown to be associated with human apraxia of speech, dyslexia, and stuttering [^38–55^](https://paperpile.com/c/Nrmxau/DNfXa+XuMCN+c0i8O+qWV69+pHDCf+u4cri+ewh7O+JSSnm+EHnai+BylC6+TMsm7+VtWQB+wkvEt+8rIFz+9ojWw+qfrmu+tw19F+7qTIt) were chosen. In order to select for likely regulatory enhancers while excluding promoters, candidate regulatory enhancers within 200,000 bp of transcription start sites (TSSs) of genes in this set were included, while those within 5,000 bp of TSSs of this gene set were excluded. This resulted in a set of 23 candidate enhancers with orthologs in up to 7 species [human (hg38), chimpanzee (panTro4), rhesus macaque (rheMac8), house mouse (mm10), Egyptian fruit bat (Raegyp2), little brown bat (myoLuc2), and zebra finch (taeGut2)]. This species set includes 3 confirmed vocal learners: human [^56^](https://paperpile.com/c/Nrmxau/sdnL2), Egyptian fruit bat [^57,58^](https://paperpile.com/c/Nrmxau/xhOEr+RK2WU), and Zebra finch [^59^](https://paperpile.com/c/Nrmxau/43yHC), amounting to a collective of 144 candidate enhancer sequences associated with vocal learning. Sequence orthologs identified across species with BLAT [^35^](https://paperpile.com/c/Nrmxau/XPZ8e) were aligned using PRANK [^60^](https://paperpile.com/c/Nrmxau/piYKd) to select the 120 bp segment with the maximal cross-species alignment.

**Experimental Design**

Plasmid Design Parameters

The new MPRA plasmid construct pAAV-Hsp68-nls/mCherry-MPRAe (pAAV-MPRAe) was synthesized based on the STARR-seq expression vectors as previously described [^2^](https://paperpile.com/c/Nrmxau/oTeqt) using VectorBuilder Cloning Services. The vector comprises of AAV2 inverted terminal repeats (ITRs) designed for utilization with the PHP.eB packaging system [^61^](https://paperpile.com/c/Nrmxau/WN6f6) or other AAV2 serotypes, along with an Hsp68 minimal promoter. The minimal promoter includes cloning sites at each end, allowing for the potential modification of the minimal promoter in future projects if desired. If multiple minimal promoters are necessary, the minimal promoter DNA barcode site can be changed by site mutagenesis (or other means) to allow for the combination of different minimal promoters within one plasmid library. The plasmid also contains a synthetic intron, mCherry reporter tagged with two SV40 Large T antigen nuclear localization signals (PKKKRKVED) on both N- and C- terminals, and a chloramphenicol resistance cassette surrounded by cloning sites used for the drop-in of the MPRA library. The Nextera Transposase binding sequences (Illumina) are located at both ends of the plasmid’s MPRA insert cloning sites, allowing for the amplification of future barcode libraries using Illumina Nextera index primers (**Supplemental Figure 1**).

Adeno-Associated Virus Packaging and Titration

Adeno-associated virus (AAV) was produced through co-transfecting of pAAV-MPRAct or pAAV-MPRAi plasmid libraries along with packaging vectors tTA- iCAP-PHP.eB (pUCmini-iCAP-PHP.eB) and pHelper into AAVpro 293T cells (Takara, #632273) using Linear 25kDa Polyethylenimine (PEI; Polysciences, #23966-2).The packaging vectors, pUCmini-iCAP-PHP.eB, and pHelper, was a kindly gifted from Vivianna Gradinaru. (<http://addgene.org/>; 103005; RRID: [Addgene_103005](https://scicrunch.org/resolver/Addgene_103005)) [^62^](https://paperpile.com/c/Nrmxau/YTxz). The media was replaced with fresh media approximately 12-18 hours post-transfection. Transfected 293T cells were cultured and 3 days post-transfection media supernatant was collected, stored at 4˚C, and replaced with fresh media. Five days post-transfection, media supernatant was collected and combined with the 3-day media supernatant. Subsequently, AAV particles were precipitated from the combined media supernatant overnight at 4˚C using 4% polyethylene glycol (PEG 8000, Sigma #P2139) in 2.5M NaCl (Thermo Fisher Scientific, #AAJ2161836). Transfected cells were collected 5 days post-transfection and centrifuged at 2,000 x g. Next, the cell pellet was lysed by resuspending the cell pellet in SAN buffer (40 mM Tris, 500 mM NaCl, 2 mM MgCl2 pH 8.0) containing Salt Activated Nuclease at 20 U/mL (SAN enzyme; Arcticzymes, #70900-201), followed by incubation at 37˚C for 1 hour with intermittent vortexing and subsequently frozen at -80˚C. To harvest the AAV virus particles from the media supernatant, 6 days post-transfection the PEG precipitated media supernatant was centrifuged at 4,000 x g and the pellets were lysed by resuspending in SAN buffer with SAN enzyme at 37˚C for 30 minutes with intermittent vortexing and subsequently store at -80˚C overnight. Next, the cell and PEG precipitated lysates were thawed for 15-30 min at 37˚C and centrifuged at 4,000 x g. AAV was collected through an iodixanol gradient via ultracentrifugation at 350,000 x g (15%, 25%, 40%, 60%; Optiprep, Sigma, #D1556-250ML) [^63^](https://paperpile.com/c/Nrmxau/u2DEN). The 40-60% interface was collected and diluted with sterile Phosphate Buffer Saline (PBS) + non-ionic detergent pluronic F-68 (Thermofisher, #24040032). Viruses were concentrated and purified using Amicon Ultra 15 kDa filter (Fisher Scientific, #UFC910096). The purified viruses were eluted in sterile PBS and filtered through a 0.22 µm syringe filter. The virus was titered using the AAVpro Titration Kit (TakaraClontech, #6233), aliquoted into LoBind tubes (Eppendorf, cat #0030108434), and stored it at −80°C until injection.

**Computational Analyses**

Processing and filtering MPRA library data

Across each of the sequenced samples (**Supplementary Table 3**), the barcode reads were counted at the DNA and RNA levels using the program arrayProc.2.1.1.py. Close matches to the designed restriction enzyme site were searched for, followed by a search for matches to the designed barcode in the neighboring bases. The number of reads with the barcode at the DNA (**Supplementary Table 4**) and RNA (**Supplementary Table 5**) level was calculated for each sample. Additionally, the proportion of those reads that include a restriction enzyme site and recognizable barcode was calculated. The library information, sample information and the barcode counts per sample were read, processed and formatted. A number of control metrics, in addition to the proportion of detected barcodes, were calculated for each sample at both DNA and RNA level. It is suspected that, in samples where the enhancer is not active, the expression levels will be low, which results in a drop-out in RNA barcode detection. Another potential cause of drop-outs in sample RNA barcodes is low sample quality. Indeed, in certain samples the amount of RNA barcodes was low, suggesting that the drop-out in these samples could be high. To identify these potential drop-outs, the enhancer RNA-DNA ratios were compared in each sample. The results showed that, in some samples, the RNA barcode levels were very low despite high DNA barcode counts (**Supplementary Figure 6A**). In some other samples, very few enhancers showed lower than expected RNA counts (**Supplementary Figure 6B**). The potential drop-out tendency is summarized with an “outlier score.” This “outlier score” is calculated as negative one tenth the number of log2 (candidate enhancer RNA counts/candidate enhancer DNA counts) < 0.3. This tends to be lower when the sample quality is lower (**Supplementary Table 3**). Based on this outlier score, samples from tissues with low amounts of RNA were excluded. The removed tissues (hypothalamus, muscle, kidney, lung, heart, ovaries, testes) most likely had low RNA quantities because only a few enhancers were designed for and active in these tissues, while the majority were inactive. For this reason, the partial frontal cortex (PFC) samples were also removed. Additionally, several individual samples were eliminated based on the “outlier score”, including the striatum for animal 5 (**Supplementary Figure 6B**) and the hippocampus from animal 6. Moreover, the RNA from HMC3 cell sample C was excluded due to low experimentally measured RNA quality and subsequent low correlation with RNA barcodes measured in comparison to other HMC3 samples. At the DNA level, the striatum sample for animal 2 (**Supplementary Figure 6A**) was eliminated due to substantially fewer reads (million reads=2.1 << median=21.6). Finally, HMC3 sample E was removed due to poor DNA correlation with all other samples (R=0.81 << median R=0.95).

**REFERENCES**

1. [Kheradpour, P., Ernst, J., Melnikov, A., Rogov, P., Wang, L., Zhang, X., Alston, J., Mikkelsen, T.S., and Kellis, M. (2013). Systematic dissection of regulatory motifs in 2000 predicted human enhancers using a massively parallel reporter assay. Genome Res. *23*, 800–811.](http://paperpile.com/b/Nrmxau/2EZ6t)

2. [Nguyen, T.A., Jones, R.D., Snavely, A.R., Pfenning, A.R., Kirchner, R., Hemberg, M., and Gray, J.M. (2016). High-throughput functional comparison of promoter and enhancer activities. Genome Res. *26*, 1023–1033.](http://paperpile.com/b/Nrmxau/oTeqt)

3. [Lee, J.W., Foo, C.S., Kim, D., Boley, N., and Kundaje, A. (2017). ATAC-Seq / DNase-Seq Pipeline.](http://paperpile.com/b/Nrmxau/Kle2e) <https://github.com/kundajelab/atac_dnase_pipelines>[.](http://paperpile.com/b/Nrmxau/Kle2e)

4. [Waterston, R.H., Lindblad-Toh, K., Birney, E., Rogers, J., Abril, J.F., Agarwal, P., Agarwala, R., Ainscough, R., Alexandersson, M., An, P., et al. (2002). Initial sequencing and comparative analysis of the mouse genome. Nature *420*, 520–562.](http://paperpile.com/b/Nrmxau/klRcI)

5. [Li, Q., Brown, J.B., Huang, H., and Bickel, P.J. (2011). Measuring reproducibility of high-throughput experiments. Ann. Appl. Stat. *5*, 1752–1779.](http://paperpile.com/b/Nrmxau/MTwwq)

6. [Srinivasan, C., Phan, B.N., Lawler, A.J., Ramamurthy, E., Kleyman, M., Brown, A.R., Kaplow, I.M., Wirthlin, M.E., and Pfenning, A.R. (2020). Addiction-associated genetic variants implicate brain cell type- and region-specific cis-regulatory elements in addiction neurobiology.](http://paperpile.com/b/Nrmxau/7JRop) [10.1101/2020.09.29.318329](http://dx.doi.org/10.1101/2020.09.29.318329)[.](http://paperpile.com/b/Nrmxau/7JRop)

7. [Stamatoyannopoulos, J.A., Snyder, M., Hardison, R., Ren, B., Gingeras, T., Gilbert, D.M., Groudine, M., Bender, M., Kaul, R., Canfield, T., et al. (2012). An encyclopedia of mouse DNA elements (Mouse ENCODE). Genome Biology *13*, 418.](http://paperpile.com/b/Nrmxau/GCJNe)

8. [ENCODE Project Consortium (2012). An integrated encyclopedia of DNA elements in the human genome. Nature *489*, 57–74.](http://paperpile.com/b/Nrmxau/pOHyN)

9. [Davis, C.A., Hitz, B.C., Sloan, C.A., Chan, E.T., Davidson, J.M., Gabdank, I., Hilton, J.A., Jain, K., Baymuradov, U.K., Narayanan, A.K., et al. (2018). The Encyclopedia of DNA elements (ENCODE): data portal update. Nucleic Acids Res. *46*, D794–D801.](http://paperpile.com/b/Nrmxau/zMDjF)

10. [Quinlan, A.R., and Hall, I.M. (2010). BEDTools: a flexible suite of utilities for comparing genomic features. Bioinformatics *26*, 841–842.](http://paperpile.com/b/Nrmxau/sJlSr)

11. [Frankish, A., Diekhans, M., Ferreira, A.M., Johnson, R., Jungreis, I., Loveland, J., Mudge, J.M., Sisu, C., Wright, J., Armstrong, J., et al. (2019). GENCODE reference annotation for the human and mouse genomes. Nucleic Acids Res. *47*, D766–D773.](http://paperpile.com/b/Nrmxau/GD5Lm)

12. [Love, M.I., Huber, W., and Anders, S. (2014). Moderated estimation of fold change and dispersion for RNA-seq data with DESeq2. Genome Biol. *15*, 550.](http://paperpile.com/b/Nrmxau/wY1pz)

13. [Bailey, T.L., Boden, M., Buske, F.A., Frith, M., Grant, C.E., Clementi, L., Ren, J., Li, W.W., and Noble, W.S. (2009). MEME Suite: Tools for motif discovery and searching. Nucleic Acids Res. *37*, W202–W208.](http://paperpile.com/b/Nrmxau/5Nvch)

14. [Telese, F., Ma, Q., Perez, P.M., Notani, D., Oh, S., Li, W., Comoletti, D., Ohgi, K.A., Taylor, H., and Rosenfeld, M.G. (2015). LRP8-Reelin-Regulated Neuronal Enhancer Signature Underlying Learning and Memory Formation. Neuron *86*, 696–710.](http://paperpile.com/b/Nrmxau/8YXZG)

15. [Lee, J.W., Boley, N., and Kundaje, A. (2016). AQUAS TF and histone ChIP-seq pipeline.](http://paperpile.com/b/Nrmxau/NRzHL)

16. [Marinov, G.K., Kundaje, A., Park, P.J., and Wold, B.J. (2014). Large-Scale Quality Analysis of Published ChIP-seq Data. G3: Genes|Genomes|Genetics *4*, 209–223.](http://paperpile.com/b/Nrmxau/eA1wl)

17. [Grant, C.E., Bailey, T.L., and Noble, W.S. (2011). FIMO: Scanning for occurrences of a given motif. Bioinformatics *27*, 1017–1018.](http://paperpile.com/b/Nrmxau/Q8cbH)

18. [Weirauch, M.T., Yang, A., Albu, M., Cote, A.G., Montenegro-Montero, A., Drewe, P., Najafabadi, H.S., Lambert, S.A., Mann, I., Cook, K., et al. (2014). Determination and inference of eukaryotic transcription factor sequence specificity. Cell *158*, 1431–1443.](http://paperpile.com/b/Nrmxau/84RII)

19. [Gerstein, M.B., Kundaje, A., Hariharan, M., Landt, S.G., Yan, K.-K., Cheng, C., Mu, X.J., Khurana, E., Rozowsky, J., Alexander, R., et al. (2012). Architecture of the human regulatory network derived from ENCODE data. Nature *489*, 91–100.](http://paperpile.com/b/Nrmxau/F3TzZ)

20. [Vermunt, M.W., Tan, S.C., Castelijns, B., Geeven, G., Reinink, P., de Bruijn, E., Kondova, I., Persengiev, S., Bontrop, R., Cuppen, E., et al. (2016). Epigenomic annotation of gene regulatory alterations during evolution of the primate brain. Nat. Neurosci. *19*, 494–503.](http://paperpile.com/b/Nrmxau/3YMkS)

21. [Villar, D., Berthelot, C., Aldridge, S., Rayner, T.F., Lukk, M., Pignatelli, M., Park, T.J., Deaville, R., Erichsen, J.T., Jasinska, A.J., et al. (2015). Enhancer evolution across 20 mammalian species. Cell *160*, 554–566.](http://paperpile.com/b/Nrmxau/k4eKO)

22. [Liao, Y., Smyth, G.K., and Shi, W. (2014). featureCounts: an efficient general purpose program for assigning sequence reads to genomic features. Bioinformatics *30*, 923–930.](http://paperpile.com/b/Nrmxau/8Vggq)

23. [Kent, W.J., Sugnet, C.W., Furey, T.S., Roskin, K.M., Pringle, T.H., Zahler, A.M., and Haussler, D. (2002). The human genome browser at UCSC. Genome Res. *12*, 996–1006.](http://paperpile.com/b/Nrmxau/n1FvP)

24. [Rosenbloom, K.R., Armstrong, J., Barber, G.P., Casper, J., Clawson, H., Diekhans, M., Dreszer, T.R., Fujita, P.A., Guruvadoo, L., Haeussler, M., et al. (2015). The UCSC Genome Browser database: 2015 update. Nucleic Acids Res. *43*, D670–D681.](http://paperpile.com/b/Nrmxau/Wd20b)

25. [Kent, W.J., Baertsch, R., Hinrichs, A., Miller, W., and Haussler, D. (2003). Evolution’s cauldron: duplication, deletion, and rearrangement in the mouse and human genomes. Proc. Natl. Acad. Sci. U. S. A. *100*, 11484–11489.](http://paperpile.com/b/Nrmxau/YoR7I)

26. [Zoonomia Consortium (2020). A comparative genomics multitool for scientific discovery and conservation. Nature *587*, 240–245.](http://paperpile.com/b/Nrmxau/7GT85)

27. [Partha, R., Chauhan, B.K., Ferreira, Z., Robinson, J.D., Lathrop, K., Nischal, K.K., Chikina, M., and Clark, N.L. (2017). Subterranean mammals show convergent regression in ocular genes and enhancers, along with adaptation to tunneling. Elife *6*, e25884.](http://paperpile.com/b/Nrmxau/hTGK2)

28. [Pollard, K.S., Hubisz, M.J., Rosenbloom, K.R., and Siepel, A. (2010). Detection of nonneutral substitution rates on mammalian phylogenies. Genome Res. *20*, 110–121.](http://paperpile.com/b/Nrmxau/DrrCT)

29. [Christmas, M.J., Kaplow, I.M., Genereux, D.P., Dong, M.X., Hughes, G.M., Li, X., Sullivan, P.F., Hindle, A.G., Andrews, G., Armstrong, J.C., et al. (2023). Evolutionary constraint and innovation across hundreds of placental mammals. Science *380*, eabn3943.](http://paperpile.com/b/Nrmxau/eoVZi)

30. [Sullivan, P., and Zoonomia Consortium Evolutionary constraint uniquely informs human disease mechanism. Preprint.](http://paperpile.com/b/Nrmxau/vJj5d)

31. [International Human Genome Sequencing Consortium (2004). Finishing the euchromatic sequence of the human genome. Nature *431*, 931–945.](http://paperpile.com/b/Nrmxau/ddkv0)

32. [Warren, W.C., Clayton, D.F., Ellegren, H., Arnold, A.P., Hillier, L.W., Künstner, A., Searle, S., White, S., Vilella, A.J., Fairley, S., et al. (2010). The genome of a songbird. Nature *464*, 757–762.](http://paperpile.com/b/Nrmxau/fulzH)

33. [Gibbs, R.A., Rogers, J., Katze, M.G., Bumgarner, R., Weinstock, G.M., Mardis, E.R., Remington, K.A., Strausberg, R.L., Venter, J.C., Wilson, R.K., et al. (2007). Evolutionary and biomedical insights from the rhesus macaque genome. Science *316*, 222–234.](http://paperpile.com/b/Nrmxau/o0YsX)

34. [Pavlovich, S.S., Lovett, S.P., Koroleva, G., Guito, J.C., Arnold, C.E., Nagle, E.R., Kulcsar, K., Lee, A., Thibaud-Nissen, F., Hume, A.J., et al. (2018). The Egyptian Rousette Genome Reveals Unexpected Features of Bat Antiviral Immunity. Cell *173*, 1098–1110.e18.](http://paperpile.com/b/Nrmxau/9p0yA)

35. [Kent, W.J. (2002). BLAT--the BLAST-like alignment tool. Genome Res. *12*, 656–664.](http://paperpile.com/b/Nrmxau/XPZ8e)

36. [Roadmap Epigenomics Consortium, Kundaje, A., Meuleman, W., Ernst, J., Bilenky, M., Yen, A., Heravi-Moussavi, A., Kheradpour, P., Zhang, Z., Wang, J., et al. (2015). Integrative analysis of 111 reference human epigenomes. Nature *518*, 317–330.](http://paperpile.com/b/Nrmxau/CLNpf)

37. [Kaplow, I.M., Schaffer, D.E., Wirthlin, M.E., Lawler, A.J., Brown, A.R., Kleyman, M., and Pfenning, A.R. (2022). Inferring mammalian tissue-specific regulatory conservation by predicting tissue-specific differences in open chromatin. BMC Genomics *23*, 291.](http://paperpile.com/b/Nrmxau/f57O6)

38. [Lai, C.S., Fisher, S.E., Hurst, J.A., Vargha-Khadem, F., and Monaco, A.P. (2001). A forkhead-domain gene is mutated in a severe speech and language disorder. Nature *413*, 519–523.](http://paperpile.com/b/Nrmxau/DNfXa)

39. [Hannula-Jouppi, K., Kaminen-Ahola, N., Taipale, M., Eklund, R., Nopola-Hemmi, J., Kääriäinen, H., and Kere, J. (2005). The axon guidance receptor gene ROBO1 is a candidate gene for developmental dyslexia. PLoS Genet. *1*, e50.](http://paperpile.com/b/Nrmxau/XuMCN)

40. [Newbury, D.F., Winchester, L., Addis, L., Paracchini, S., Buckingham, L.-L., Clark, A., Cohen, W., Cowie, H., Dworzynski, K., Everitt, A., et al. (2009). CMIP and ATP2C2 modulate phonological short-term memory in language impairment. Am. J. Hum. Genet. *85*, 264–272.](http://paperpile.com/b/Nrmxau/c0i8O)

41. [Konopka, G., Bomar, J.M., Winden, K., Coppola, G., Jonsson, Z.O., Gao, F., Peng, S., Preuss, T.M., Wohlschlegel, J.A., and Geschwind, D.H. (2009). Human-specific transcriptional regulation of CNS development genes by FOXP2. Nature *462*, 213–217.](http://paperpile.com/b/Nrmxau/qWV69)

42. [König, I.R., Schumacher, J., Hoffmann, P., Kleensang, A., Ludwig, K.U., Grimm, T., Neuhoff, N., Preis, M., Roeske, D., Warnke, A., et al. (2011). Mapping for dyslexia and related cognitive trait loci provides strong evidence for further risk genes on chromosome 6p21. Am. J. Med. Genet. B Neuropsychiatr. Genet. *156B*, 36–43.](http://paperpile.com/b/Nrmxau/pHDCf)

43. [Horn, D., Kapeller, J., Rivera-Brugués, N., Moog, U., Lorenz-Depiereux, B., Eck, S., Hempel, M., Wagenstaller, J., Gawthrope, A., Monaco, A.P., et al. (2010). Identification of FOXP1 deletions in three unrelated patients with mental retardation and significant speech and language deficits. Hum. Mutat. *31*, E1851–E1860.](http://paperpile.com/b/Nrmxau/u4cri)

44. [Filges, I., Shimojima, K., Okamoto, N., Röthlisberger, B., Weber, P., Huber, A.R., Nishizawa, T., Datta, A.N., Miny, P., and Yamamoto, T. (2011). Reduced expression by SETBP1 haploinsufficiency causes developmental and expressive language delay indicating a phenotype distinct from Schinzel--Giedion syndrome. J. Med. Genet. *48*, 117–122.](http://paperpile.com/b/Nrmxau/ewh7O)

45. [Roeske, D., Ludwig, K.U., Neuhoff, N., Becker, J., Bartling, J., Bruder, J., Brockschmidt, F.F., Warnke, A., Remschmidt, H., Hoffmann, P., et al. (2011). First genome-wide association scan on neurophysiological endophenotypes points to trans-regulation effects on SLC2A3 in dyslexic children. Mol. Psychiatry *16*, 97–107.](http://paperpile.com/b/Nrmxau/JSSnm)

46. [Bacon, C., and Rappold, G.A. (2012). The distinct and overlapping phenotypic spectra of FOXP1 and FOXP2 in cognitive disorders. Hum. Genet. *131*, 1687–1698.](http://paperpile.com/b/Nrmxau/EHnai)

47. [Thevenon, J., Callier, P., Andrieux, J., Delobel, B., David, A., Sukno, S., Minot, D., Mosca Anne, L., Marle, N., Sanlaville, D., et al. (2013). 12p13.33 microdeletion including ELKS/ERC1, a new locus associated with childhood apraxia of speech. Eur. J. Hum. Genet. *21*, 82–88.](http://paperpile.com/b/Nrmxau/BylC6)

48. [Amarillo, I.E., Li, W.L., Li, X., Vilain, E., and Kantarci, S. (2014). De novo single exon deletion of AUTS2 in a patient with speech and language disorder: a review of disrupted AUTS2 and further evidence for its role in neurodevelopmental disorders. Am. J. Med. Genet. A *164A*, 958–965.](http://paperpile.com/b/Nrmxau/TMsm7)

49. [Peter, B., Matsushita, M., Oda, K., and Raskind, W. (2014). De novo microdeletion of BCL11A is associated with severe speech sound disorder. Am. J. Med. Genet. A *164A*, 2091–2096.](http://paperpile.com/b/Nrmxau/VtWQB)

50. [Namjou, B., Marsolo, K., Caroll, R.J., Denny, J.C., Ritchie, M.D., Verma, S.S., Lingren, T., Porollo, A., Cobb, B.L., Perry, C., et al. (2014). Phenome-wide association study (PheWAS) in EMR-linked pediatric cohorts, genetically links PLCL1 to speech language development and IL5-IL13 to Eosinophilic Esophagitis. Front. Genet. *5*, 401.](http://paperpile.com/b/Nrmxau/wkvEt)

51. [Turner, S.J., Mayes, A.K., Verhoeven, A., Mandelstam, S.A., Morgan, A.T., and Scheffer, I.E. (2015). GRIN2A: an aptly named gene for speech dysfunction. Neurology *84*, 586–593.](http://paperpile.com/b/Nrmxau/8rIFz)

52. [Chen, X.S., Reader, R.H., Hoischen, A., Veltman, J.A., Simpson, N.H., Francks, C., Newbury, D.F., and Fisher, S.E. (2017). Next-generation DNA sequencing identifies novel gene variants and pathways involved in specific language impairment. Sci. Rep. *7*, 46105.](http://paperpile.com/b/Nrmxau/9ojWw)

53. [Senkevich, K., Miliukhina, I., Shadenkov, V., Gracheva, E., Maria, B., Kulabukhova, D., Emelyanov, A., and Pchelina, S. (2018). Associations of genetic variants in COMT, BDNF, SNCA, MAPT genes with cognitive impairment in Parkinson’s disease. (P6.090). Neurology *90*.](http://paperpile.com/b/Nrmxau/qfrmu)

54. [Eising, E., Carrion-Castillo, A., Vino, A., Strand, E.A., Jakielski, K.J., Scerri, T.S., Hildebrand, M.S., Webster, R., Ma, A., Mazoyer, B., et al. (2019). A set of regulatory genes co-expressed in embryonic human brain is implicated in disrupted speech development. Mol. Psychiatry *24*, 1065–1078.](http://paperpile.com/b/Nrmxau/tw19F)

55. [Kielb, S., Kisanuki, Y.Y., and Dawson, E. (2021). Neuropsychological profile associated with an alpha-synuclein gene (SNCA) duplication. Clin. Neuropsychol., 1–12.](http://paperpile.com/b/Nrmxau/7qTIt)

56. [Jarvis, E.D. (2019). Evolution of vocal learning and spoken language. Science *366*, 50–54.](http://paperpile.com/b/Nrmxau/sdnL2)

57. [Prat, Y., Taub, M., and Yovel, Y. (2015). Vocal learning in a social mammal: Demonstrated by isolation and playback experiments in bats. Preprint,](http://paperpile.com/b/Nrmxau/xhOEr) [10.1126/sciadv.1500019](http://dx.doi.org/10.1126/sciadv.1500019)[10.1126/sciadv.1500019](http://dx.doi.org/10.1126/sciadv.1500019)[.](http://paperpile.com/b/Nrmxau/xhOEr)

58. [Genzel, D., Desai, J., Paras, E., and Yartsev, M.M. (2019). Long-term and persistent vocal plasticity in adult bats. Nat. Commun. *10*, 3372.](http://paperpile.com/b/Nrmxau/RK2WU)

59. [Zeigler, H.P., and Marler, P. eds. (2008). Neuroscience of birdsong. *550*.](http://paperpile.com/b/Nrmxau/43yHC)

60. [Löytynoja, A., and Goldman, N. (2008). Phylogeny-aware gap placement prevents errors in sequence alignment and evolutionary analysis. Science *320*, 1632–1635.](http://paperpile.com/b/Nrmxau/piYKd)

61. [Deverman, B.E., Pravdo, P.L., Simpson, B.P., Kumar, S.R., Chan, K.Y., Banerjee, A., Wu, W.-L., Yang, B., Huber, N., Pasca, S.P., et al. (2016). Cre-dependent selection yields AAV variants for widespread gene transfer to the adult brain. Nat. Biotechnol. *34*, 204–209.](http://paperpile.com/b/Nrmxau/WN6f6)

62. [Chan, K.Y., Jang, M.J., Yoo, B.B., Greenbaum, A., Ravi, N., Wu, W.-L., Sánchez-Guardado, L., Lois, C., Mazmanian, S.K., Deverman, B.E., et al. (2017). Engineered AAVs for efficient noninvasive gene delivery to the central and peripheral nervous systems. Nat. Neurosci. *20*, 1172–1179.](http://paperpile.com/b/Nrmxau/YTxz)

63. [Lock, M., Alvira, M., Vandenberghe, L.H., Samanta, A., Toelen, J., Debyser, Z., and Wilson, J.M. (2010). Rapid, simple, and versatile manufacturing of recombinant adeno-associated viral vectors at scale. Hum. Gene Ther. *21*, 1259–1271.](http://paperpile.com/b/Nrmxau/u2DEN)
